# Mesenchymal-endothelial transition-derived cells as a potential new regulatory target for cardiac hypertrophy

**DOI:** 10.1038/s41598-020-63671-8

**Published:** 2020-04-20

**Authors:** Wenyan Dong, Ruiqi Li, Haili Yang, Yan Lu, Longhai Zhou, Lei Sun, Dianliang Wang, Jinzhu Duan

**Affiliations:** 1 Heart Center and Institute of Pediatrics, Guangzhou Women and Children’s Medical Center, Guangzhou Medical University, Guangzhou, 510120 China; 2 Department of Pathology, University of Washington, Seattle, 98109 WA USA; 3 Stem Cell and Tissue Engineering Research Laboratory, Department of Pharmacy, PLA Rocket Force Characteristic Medical Center, Beijing, 100088 China

**Keywords:** Stem cells, Cardiology

## Abstract

The role of Mesenchymal-endothelial transition (MEndoT) in cardiac hypertrophy is unclear. To determine the difference between MEndoT-derived and coronary endothelial cells is essential for understanding the revascularizing strategy in cardiac repair. Using lineage tracing we demonstrated that MEndoT-derived cells exhibit highly heterogeneous which were characterized with highly expression of endothelial markers such as vascular endothelial cadherin(VECAD) and occludin but low expression of Tek receptor tyrosine kinase(Tek), isolectin B4, endothelial nitric oxide synthase(eNOS), von Willebrand factor(vWF), and CD31 after cardiac hypertrophy. RNA-sequencing showed altered expression of fibroblast lineage commitment genes in fibroblasts undergoing MEndoT. Compared with fibroblasts, the expression of p53 and most endothelial lineage commitment genes were upregulated in MEndoT-derived cells; however, the further analysis indicated that MEndoT-derived cells may represent an endothelial-like cell sub-population. Loss and gain function study demonstrated that MEndoT-derived cells are substantial sources of neovascularization, which can be manipulated to attenuate cardiac hypertrophy and preserve cardiac function by improving the expression of endothelial markers in MEndoT-derived cells. Moreover, fibroblasts undergoing MEndoT showed significantly upregulated anti-hypertrophic factors and downregulated pro-hypertrophic factors. Therefore MEndoT-derived cells are an endothelial-like cell population that can be regulated to treat cardiac hypertrophy by improving neovascularization and altering the paracrine effect of fibroblasts.

## Introduction

Myocardial hypertrophy is an adaptive response to pressure and volume overload, which is an early milestone during the clinical course of heart failure and an important risk factor for subsequent cardiac morbidity and mortality^1^. Nonmyocytes in the interstitium, including vascular endothelial and fibroblast, appear to be crucially involved in the myocardial response to external and internal stress^2^. Despite its importance in the development of heart failure, the interplay between fibroblast, angiogenesis, and cardiomyocytes in cardiac hypertrophy is still not well understood.

Mesenchymal-endothelial transition (MEndoT) is a phenomena recently identified in ischemia reperfusion and modulation of MEndoT represents a potential therapy for cardiac repair by rapidly increasing vascularity^3^. Several publications have confirmed or corroborated the existence of MEndoT, or provided additional observations of its effects^4,5^. However, the role of MEndoT-derived cells in cardiac hypertrophy is unknown.

Identification of the expression of characteristic endothelial cell markers is essential for understanding the function of MEndoT-derived cells in cardiovascular disease, which can help us figuring out how to regulate MEndoT-derived cells into specific endothelial cell population for therapeutics interventions. Endothelium exhibits a significant heterogeneity which differs from organ to organ, and there are differences between macrovascular and microvascular endothelial cells^6–8^. Many endothelial cell markers are available, which exhibit preferential staining for different endothelial cell populations^9^. Cardiac Microvascular Endothelial Cells(CMEC) are increasingly attracting attention in the fields of cardiac physiology and biology for they can exert (patho)physiological effects on the function of cardicmyocytes^8,10^. CMEC has been characterized by genetic expression of endothelial markers like CD31, von Willebrand factor(vWF), isolectin B4, endothelial nitric oxide synthase(eNOS), Tek receptor tyrosine kinase(Tek), vascular endothelial cadherin(VECAD) etc. However, the expression level among these markers in CMEC may vary according to literatures^8,10–14^. Thus, to determine the potential phenotypic properties and characteristic of MEndoT-derived cells especially relating to CMEC will provide us a guidance for specific regulation of MEndoT-derived cell in cardiac repair.

Following our previous work, after introducing multiple fibroblast and endothelial cell lineage labeling transgenic mice, we investigated the occurrence, gene expression profile, and pathophysiological role of MEndoT-derived cells in cardiac hypertrophy aimed at providing deeper insight into the characteristics of MEndoT-derived cells and advancing our understanding of MEndoT in cardiac repair.

## Results

### MEndoT-derived cells exhibit high heterogeneity with respect to the expression of endothelial cell markers after cardiac hypertrophy

Col1a2 (collagen1a2) -CreERT: R26R^tdTomato^ and TCF21 (transcription factor 21) -MerCreMer: R26R^tdTomato^ mice were generated by crossing Cre mice with lineage reporter R26R^tdTomato^ mice, where a tamoxifen inducible Cre recombinase was driven by enhancer elements of *Col1a2* and *TCF21*, respectively. The Col1a2^3,15–17^ and TCF21^18–20^ labels were reported as reliable markers to identify cardiac fibroblasts in the adult mouse heart.

We subjected Col1a2 -CreERT(or TCF21 -MerCreMer): R26R^tdTomato^ mice to sham injury or transverse aortic constriction (TAC) 5 days following cessation of tamoxifen injection and examined the injured hearts using immunofluorescence staining of endothelial cell markers. Cells expressing both the fibroblast label (tdTomato) and endothelial marker in Col1a2 -CreERT(or TCF21 -MerCreMer): R26R^tdTomato^ mice were identified as fibroblasts undergoing MEndoT.

**Figure 1:**
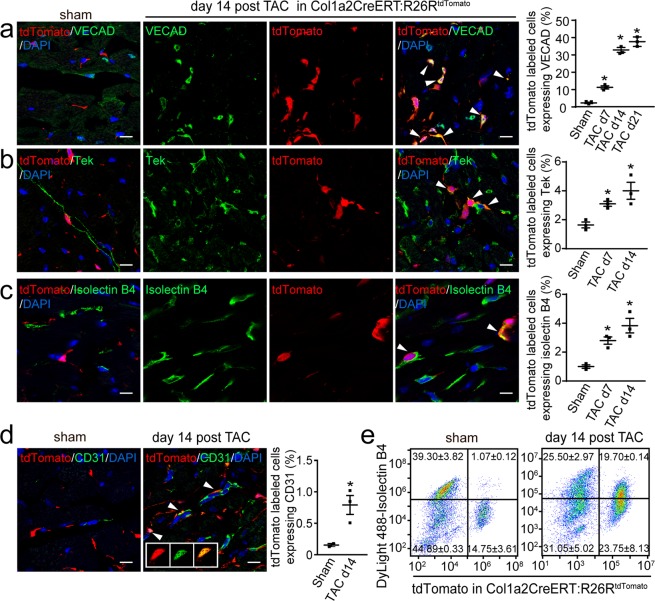
Expression of endothelial markers in mesenchymal-endothelial transition (MEndoT)-derived cells in the heart tissue of Col1a2-CreERT: R26R^tdTomato^ mice after cardiac hypertrophy. (**a-d**) Immunofluorescence staining for endothelial cell markers in heart tissue of Col1a2-CreERT: R26R^tdTomato^ mice after transverse aortic constriction (TAC). (**a**) VECAD expression and the percentage of tdTomato labeled cells expressing VECAD. (**b**) Tek expression and the percentage of tdTomato labeled cells expressing Tek. (**c**) Isolectin B4 expression and the percentage of tdTomato labeled cells expressing isolectin B4. (**d**) CD31 expression and the percentage of tdTomato labeled cells expressing CD31. (**e**) Flow cytometry of isolectin B4 14 days post sham or TAC. (All graphs show mean ± S.E.M; n = 3 animals/group, *p < 0.05, using an unpaired t-test compared with sham. Colocalization of fluorophores is indicated by arrowhead. Scale bar: 10 µm).

In Col1a2-CreERT: R26R^tdTomato^ mice, we observed that by day 14 post-TAC, 33.4 ± 1.1% of tdTomato-labeled cardiac fibroblasts expressed the endothelial marker VECAD, whereas the increase was minor at 36.6 ± 2.2% by 21 days post-TAC (Fig. 1a). Similar results were also obtained with MEndoT staining with VECAD in Angiotensin (Ang)-II-induced cardiac hypertrophy (Supplemental Fig. 1a,b). In sham-injured hearts, <1.0 ± 1% of labeled cardiac fibroblasts expressed VECAD (Fig. 1a).

We observed that after TAC, the tdTomato-labeled cardiac fibroblasts highly expressed the endothelial marker occludin at 37.2 ± 4.1% (Supplemental Fig. 1c,d) which was similar to the MEndoT-derived cells during ischemia-reperfusion injury^3^. However, expression of the endothelial marker eNOS in tdTomato-labeled cardiac fibroblasts was very low at 1.0 ± 0.1% (Supplemental Fig. 1e,f) compared to 24 ± 4% in ischemia-reperfusion injury^3^, which indicates that the characteristics of MEndoT-derived cells may varied depending on the injury model.

To further understand the characteristics of MEndoT-derived cells after TAC, we also investigated their expression of other widely used endothelial markers especially for CMEC, which play an important role in maintaining cardiomyocyte function such as isolectin B4, vWF, Tek and CD31. In Col1a2-CreERT: R26R^tdTomato^ mice 14 days post-TAC, we observed that the percentage of labeled cardiac fibroblasts expressing the endothelial markers Tek, isolectin B4 and vWF was 4 ± 0.6%, 3.5 ± 0.5% and 4.2 ± 0.4%, respectively (Fig. 1b,c and Supplemental Fig. 1g,h). However, few labeled cardiac fibroblasts expressed CD31 after TAC (Fig. 1d). The flow cytometry assay of isolated non-myocyte cells further showed that tdTomato-labeled fibroblasts expressing the endothelial marker isolectin B4 increased from 1.07 ± 0.12% after sham to 19.70 ± 0.14% 14 days post-TAC in Col1a2-CreERT: R26R^tdTomato^ mice (Fig. 1e). The difference in the fold change of isolectin B4 by immunofluorescence staining and flow cytometry (Fig. 1c vs 1e) may be due to different methodology.

To further confirm our results in Col1a2-CreERT: R26R^tdTomato^ mice, we investigated MEndoT-derived cells using another cardiac fibroblast marker-labeled mouse model, TCF21-MerCreMer: R26R^tdTomato^. Again, by day 14 post-TAC, 43.9 ± 1.8% of tdTomato-labeled cardiac fibroblasts expressed VECAD, which was slightly higher than the MEndoT-derived cells of Col1a2-CreERT: R26R^tdTomato^ mice **(**Supplemental Fig. 2a**)**. Similarly to MEndoT-derived cells of Col1a2-CreERT: R26R^tdTomato^ mice, the percentage of labeled cardiac fibroblasts expressing Tek, isolectin B4, eNOS, and CD31 in TCF21-MerCreMer: R26R^tdTomato^ after TAC was 4.3 ± 0.4%, 2.6 ± 0.2%, 2.2 ± 0.6%, and 0.8 ± 0.1%, respectively (Supplemental Fig. 2b–e). However, in contrast to the 4.2 ± 0.4% labeled cardiac fibroblasts expressing vWF in Col1a2-CreERT: R26R^tdTomato^ mice, we observed no vWF expression in labeled cardiac fibroblasts of TCF21-MerCreMer: R26R^tdTomato^ mice 14 days post-TAC (Supplemental Fig. 2f). In summary, giving the significantly increased MEndoT-derived cells from sham to injury group, and the viability among different endothelial markers, the increasement of MEndoT-derived cells should not mainly due to the expansion of a small subset of cell population following injury

### MEndoT-derived cells are another substantial source of endothelial cells after cardiac injury

Tek labeling^21,22^ was reported as a reliable marker to identify endothelial cells in the adult mouse heart. To compare MEndoT-derived and native endothelial cells in parallel, we generated Tek-CreERT: R26R^tdTomato^ mice, which were subjected to sham operations or TAC, similar to the Col1a2-CreERT: R26R^tdTomato^ mice. We observed that 15.1 ± 1.0% of the tdTomato-labeled native coronary endothelial cells expressed VECAD in sham injury mice, which then increased to 51.4 ± 3.4% 14 days post-TAC (Supplemental Fig. 3a), which was 53% higher than levels of MEndoT-derived cells after cardiac hypertrophy. However, a significant difference was observed between endothelial and MEndoT-derived cells in the expression of other endothelial cell markers such as Tek, isolectin B4, vWF, and CD31 14 days post-TAC in the heart of Tek-CreERT:R26R^tdTomato^ mice. The percentage of labeled cardiac endothelial cells expressing Tek, isolectin B4, vWF, eNOS, and CD31 was 33.4 ± 1.1%, 33.9 ± 1.4%, 22.8 ± 0.5%, 22.7 ± 1.3%, and 28.0 ± 0.7%, respectively (Supplemental Fig. 3b–f). These results showed that the endothelial cell markers were also not uniformly expressed by endothelial cells simultaneously.

Intriguingly, the Tek labeling endothelial cells expressing endothelial markers is low at the sham baseline. Considering fixation may cause some loss of antigen epitope in preparation of frozen section, we performed flow cytometry by using Tek-CreERT:R26R^tdTomato^:Tie2GFP mice, where the expression of Tek in real time will be labelled by GFP. Tyrosine kinase with immunoglobulin and epidermal growth factor homology domain-2 (Tie2) is also known as Tek. After 10 days of administration of tamoxifen and 5 days cessation, only 61% Tek-CreERT:R26R^tdTomato^ labeled endothelial cells are GFP positive (Supplemental Fig. 4). This result indicates a dynamic expression of endothelial cell surface markers in endothelial cells.

Compared with native coronary endothelial cells, the expression of these endothelial cell markers varied in MEndoT-derived cells and was low. Most of the endothelial cell markers above were highly expressed in native coronary endothelial cells and some of them play roles in endothelial cell function such as eNOS. Thus, our finding indicates that MEndoT-derived cells are more highly heterogeneous cell populations than coronary endothelial cells, and the low expression of endothelial cell markers may be due to the lack of functional maturity in most of MEndoT-derived cells.

**Figure 2:**
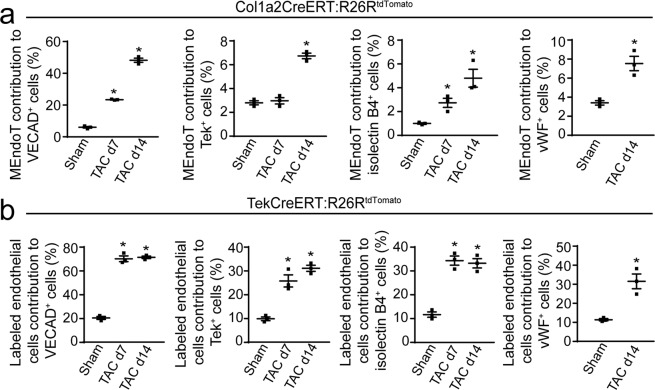
Contribution of mesenchymal-endothelial transition (MEndoT)-derived cells to neovascularization of heart tissue after cardiac hypertrophy. Contribution to neovascularization was calculated based on immunofluorescence staining for endothelial cell markers in heart tissue of Col1a2-CreERT: R26R^tdTomato^ mice for MEndoT-derived cells (**a**) and Tek-CreERT: R26R^tdTomato^ mice for labeled coronary endothelial cells (**b**). (All graphs show mean ± S.E.M; n = 3 animals/group, *p < 0.05, using an unpaired t-test compared with sham).

Comparing MEndoT-derived cells and preexisting coronary vessels in parallel is essential to understanding the significance of MEndoT-derived cells in the strategy of revascularizing damaged myocardium during cardiac repair. Based on the immunoflurescence staining data from Fig. 1, we calculated the contribution of MEndoT-derived cells to various endothelial cell populations identified by different endothelial cell markers using following formula: tdTomato^+^ endothelial cell marker^+^ cells /total endothelial cell marker^+^ cells. We observed that the contribution of MEndoT-derived cells to various endothelial cell population varied significantly among the different endothelial cell markers, which showed a high contribution of VECAD- (48.0 ± 0.9%) but low contribution of isolectin B4 (4.7 ± 0.5%), Tek (6.7 ± 0.2%), and vWF(7.4 ± 0.8%)-positive endothelial cells (Fig. 2a). Furthermore, there was low contribution of CD31- (1.8 ± 0.3%) and eNOS-positive endothelial cells in Col1a2-CreERT: R26R^tdTomato^ mice after TAC. In contrast, the prelabeled endothelial cells of the Tek-CreERT: R26R^tdTomato^ mice showed a high contribution of VECAD-, isolectin B4-, Tek-, vWF-, CD31-, and eNOS-positive endothelial cells after TAC at 71.4 ± 1.8%, 33.2 ± 4.8%, 31.1 ± 2.2%, 31.1 ± 3.5%, 28.0 ± 1.9%, and 40.6 ± 3.7%, respectively (Fig. 2b, the calculation was based on immunoflurescence staining data from Supplemental Fig. 3 by using formula as Col1a2-CreERT: R26R^tdTomato^ mice). These results demonstrated that MEndoT-derived endothelial cells are another substantial source of neovascularization during cardiac hypertrophy, which exhibited different characteristics from those of preexisting coronary endothelial cells.

### Cardiac hypertrophy-induced MEndoT-derived cells are a more endothelial-like cell sub-population

To enhance our understanding of the molecular characteristics of MEndoT-derived cells, we isolated non-myocytes from the hearts of 14 days post-sham or -TAC Col1a2-CreERT: R26R^tdTomato^ mice and performed flow-sorting using tdTomato and Brilliant Violet 421-conjugated antibodies against CD31. Although the VECAD is ideal for sorting, only the flow cytometry CD31 antibody worked in freshly isolated live endothelial cells in this study. We observed that the CD31 expression of tdTomato-labeled cardiac fibroblasts was low and accounted for 0.15 ± 0.03% and 0.11 ± 0.02% at day 14 post-TAC in Col1a2-CreERT: R26R^tdTomato^ and TCF21-MerCreMer: R26R^tdTomato^ mice, respectively (Supplemental Fig. 5a,b). In sham-injured hearts, the fraction of labeled cardiac fibroblasts expressing CD31 was <0.06 ± 0.03% (Supplemental Fig. 5a,b). In Supplemental Fig. 5a, the percentage of CD31 population decreased after TAC may be due to significantly expanded fibroblasts after injury, resulting in the dilution of total endothelial cells compared to total non-myocyte cells.

We then collect approximately 100–200 MEndoT-derived cells from each mouse (tdTomato^+^CD31^+^ labeled cells in Col1a2-CreERT: R26R^tdTomato^ mice after sham/TAC) for RNA-seq. The tdTomato-labeled fibroblasts from the sham-injured hearts of Col1a2-CreERT: R26R^tdTomato^ mice and tdTomato-labeled endothelial cells from sham-injured hearts of Tek-CreERT: R26R^tdTomato^ mice were also collected similarly to MEndoT-derived cells and served as native cardiac fibroblasts and coronary endothelial cells, respectively.

**Figure 3:**
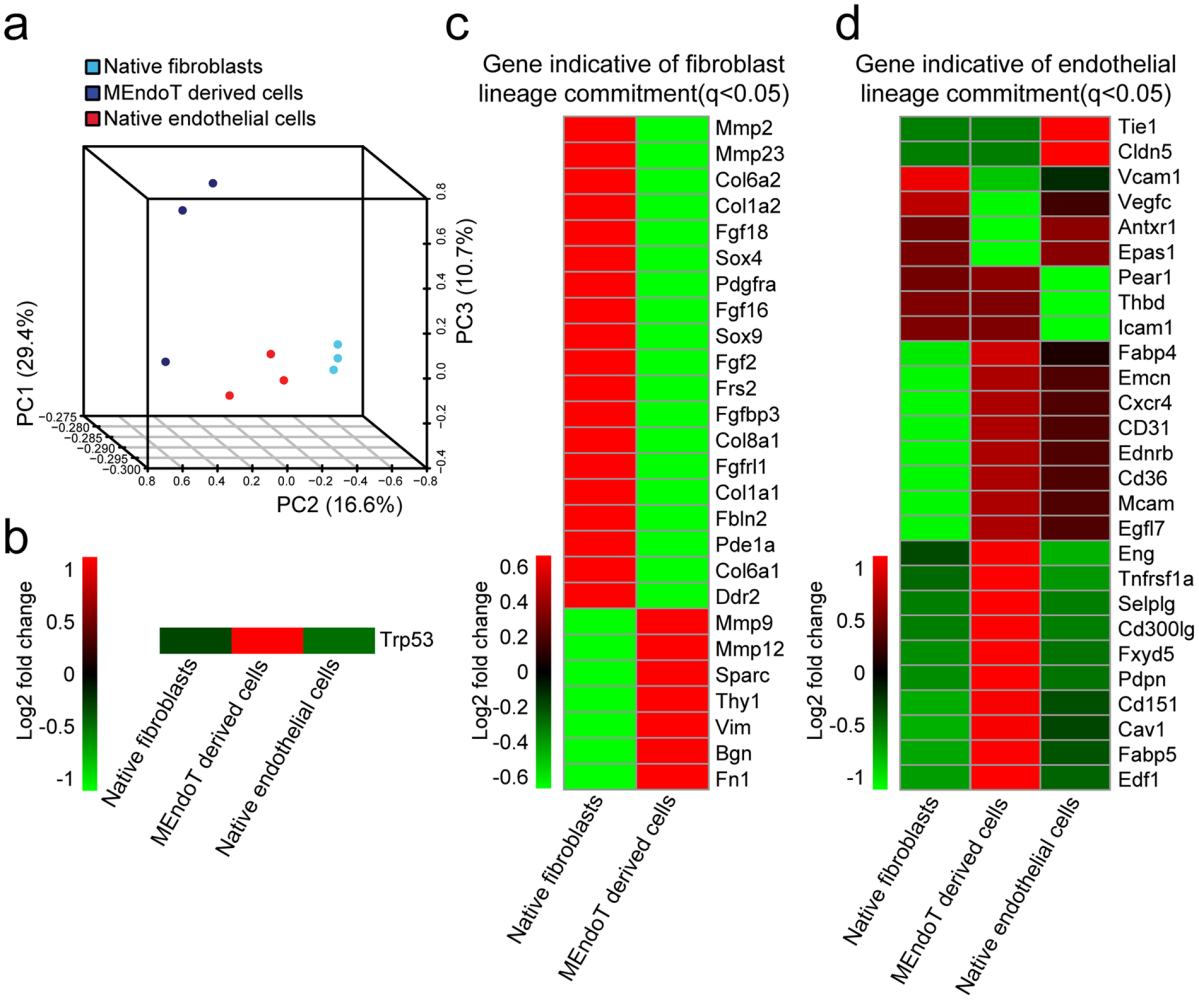
RNA sequencing (RNA-seq) assay shows mesenchymal-endothelial transition (MEndoT)-derived cells are more endothelial-like after cardiac hypertrophy but may be a different cell sub-population. Non-myocytes were isolated from hearts of 14 day post-sham or -transverse aortic constriction (TAC) in Col1a2-CreERT: R26R^tdTomato^ mice and native fibroblasts (tdTomato^+^-labeled fibroblasts after sham-injury), MEndoT-derived cells (tdTomato^+^CD31^+^-labeled cells) were collected using flow cytometry for RNA-seq. The tdTomato^+^-labeled endothelial cells from heart tissue of Tek-CreERT: R26R^tdTomato^ mice after sham injury were collected and served as native coronary endothelial cells. (**a**) Principal component analysis clustering show MEndoT-derived cells are a subset cell population with characteristics that were closer to native endothelial cells than native fibroblasts. (**b**) p53 is specifically and significantly upregulated in MEndoT-derived cells. Heatmap show significantly expressed genes indicative of (**c**) fibroblast lineage commitment and (**d**) endothelial lineage commitment (all genes in b-d have a q-value <0.05 compared with native fibroblast, n = 3 animals/group).

Principal component analysis clustering showed that MEndoT-derived cells had characteristics that were closer to those of native endothelial cells than native fibroblasts (Fig. 3a). Interestingly, RNA-seq data also showed that p53 was specifically and significantly enhanced in MEndoT-derived cells (Fig. 3b), which was consistent with our previous finding that p53 is upregulated in fibroblast cells undergoing MEndoT after ischemia reperfusion^3^.

Compared with native fibroblasts, the expression of most genes indicative of fibroblast lineage commitment were altered in MEndoT-derived cells including the widely used fibroblast-specific markers (such as Col1a2, collagen1a1 (Col1a1), discoidin domain receptor tyrosine kinase 2 (DDR2), platelet derived growth factor receptor, alpha(PDGFrα), and TCF21) and mesenchymal cells markers (such as sex determining region Y (SRY)-box 9 (sox9) and T-box 18 (Tbx18)), which were downregulated in fibroblast subjected to MEndoT (Fig. 3c, Supplemental Fig. 5c, and Supplemental Table 1). In contrast, the expression of most genes indicative of endothelial lineage commitment were upregulated in MEndoT-derived cells including widely used endothelial cell specific markers such as CD31 (PECAM1), VECAD (cdh5), occludin, FMS-like tyrosine kinase 1 (Flt1), and kinase insert domain protein receptor (Kdr) (Fig. 3d, Supplemental Fig. 5d and Supplemental Table 2).

MEndoT-derived and native endothelial cells share 501 genes compared with native fibroblast cells, and the pathway enrichment and go function assay further showed that MEndoT-derived and native endothelial cells share most common pathways and go function enrichment compared with native fibroblast cells (Supplemental Figs. 6 and 7), which again, indicated the similarity between MEndoT-derived and native endothelial cells. However, there were numerous difference between MEndoT-derived and native endothelial cells in the expression of some endothelial lineage commitment genes (Fig. 3d, Supplemental Fig. 5d and Supplemental Table 2), the KEGG pathway^23^ and the go function enrichment^24^ (Supplemental Figs. 6 and 7). These differences indicate that MEndoT-derived cells may represent a new cell sub-population that is different from prelabeled native endothelial cells.

### MEndoT occurs partly in p53-dependent manner after cardiac hypertrophy

To determine whether p53 also mediates MEndoT after cardiac hypertrophy, we generated Col1a2-CreERT: R26R^tdTomato^: p53CKO mice [Col1a2-CreERT: R26R^tdTomato^: p53^fl/fl^ mice, referred to as conditional knockout] and subjected them to TAC, and fibroblasts with p53 deficiency following tamoxifen administration were also labeled by tdTomato fluorescence.

**Figure 4:**
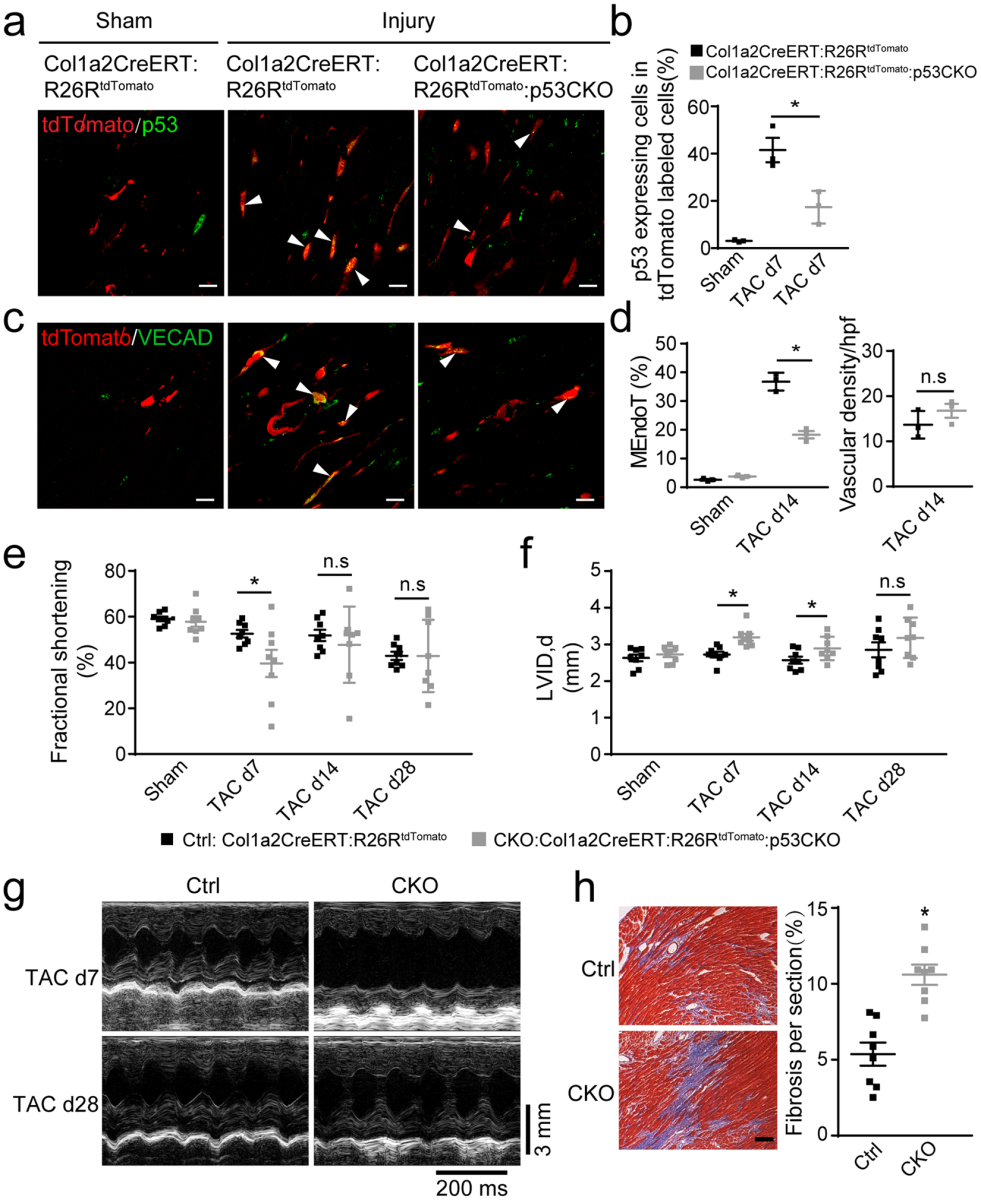
Cardiac fibroblasts adopt endothelial cell characteristics after cardiac hypertrophy in a p53-dependent manner and conditional deletion of p53 in cardiac fibroblasts leads to temporary deterioration of cardiac function after cardiac hypertrophy. Hearts of Col1a2-CreERT: R26R^tdTomato^ and Col1a2-CreERT: R26R^tdTomato^: p53CKO mice show (**a**) immunofluorescence staining of p53 7 days post-sham or -transverse aortic constriction (TAC) and (**b**) percentage of tdTomato-labeled cells expressing p53 in (**a**). (**c**) Immunofluorescence staining for endothelial markers vascular endothelial cadherin (VECAD) 14 days post-sham or -TAC. (**d**) Percentage of tdTomato-labeled cells expressing VECAD and vascular density per high-power field (hpf) in (**c**).(**e**,**f**) echocardiographic assessment of cardiac function prior to and after transverse aortic constriction (TAC, n = 8 animals/group, *p < 0.05 using ANOVA with post hoc tests). (**g**) Representative M-mode echocardiograms in (**e**,**f**). (**h**) Quantitation of fibrotic area in heart using Masson’s trichrome staining of sections isolated 5 weeks after TAC (n = 8 animals/group, Scale bar: 25 µm). All graphs show mean ± S.E.M.; n = 3 animals/group for a-d, *p < 0.05 using an unpaired t-test as indicated, n.s. not significant. Colocalization of fluorophores indicated by arrowhead. Scale bar: 10 µm.

We subjected Col1a2-CreERT: R26R^tdTomato^: p53CKO mice to sham injury or TAC 5 days following cessation of tamoxifen injection and examined the injured hearts by immunofluorescence staining of endothelial cell markers, we observed that the expression of p53 was enhanced in tdTomato-labeled fibroblast cells in Col1a2-CreERT: R26R^tdTomato^ mice after TAC, whereas p53 expression levels of tdTomato-labeled cardiac fibroblasts were significantly reduced by approximately 60% on day 7 post-TAC in p53CKO mice (Fig. 4a,b). Following the knockdown of p53, the MEndoT decreased by 40% in p53 deficient fibroblasts (Fig. 4c,d) and the contribution of MEndoT to endothelial cells decreased by 30% compared with that of intact p53 animals by day 14 post-TAC. However, we did not observe significant differences in the vascular density assay on day 14 post-TAC **(**Fig. 4d**)** and there are also no significant difference in the ratio of heart to body weight and the area of cadiomyocyte 28 days post-TAC in mice with p53-deficient fibroblasts (Supplemental Fig. 8). These observations suggest that compensatory mechanisms may have been initiated during the chronic disease.

We performed echocardiography on the hearts of the mice 7, 14, and 28 days post-TAC and observed that the fractional shortening and left ventricular internal diameter (LVID) was significantly worse only at the early days post-TAC (Fig. 4e-g) in Col1a2-CreERT: R26R^tdTomato^: p53CKO mice compared to murine hearts with intact p53. This observation also suggests that compensatory mechanisms were likely triggered during the chronic disease because of the gene-deleting efficiency or because MEndoT is partly dependent on p53. The Masson’s trichrome staining of the heart sections showed significant increase in interstitial fibrosis in p53-deficient fibroblasts 28 days post-TAC (Fig. 4h). All these data suggest that p53 also partly mediates MEndoT during cardiac hypertrophy.

### Stimulation of p53 signaling attenuates cardiac hypertrophy and preserves heart function by improving maturity of MEndoT-derived cells and altering expression of paracrine factors in fibroblasts

**Figure 5:**
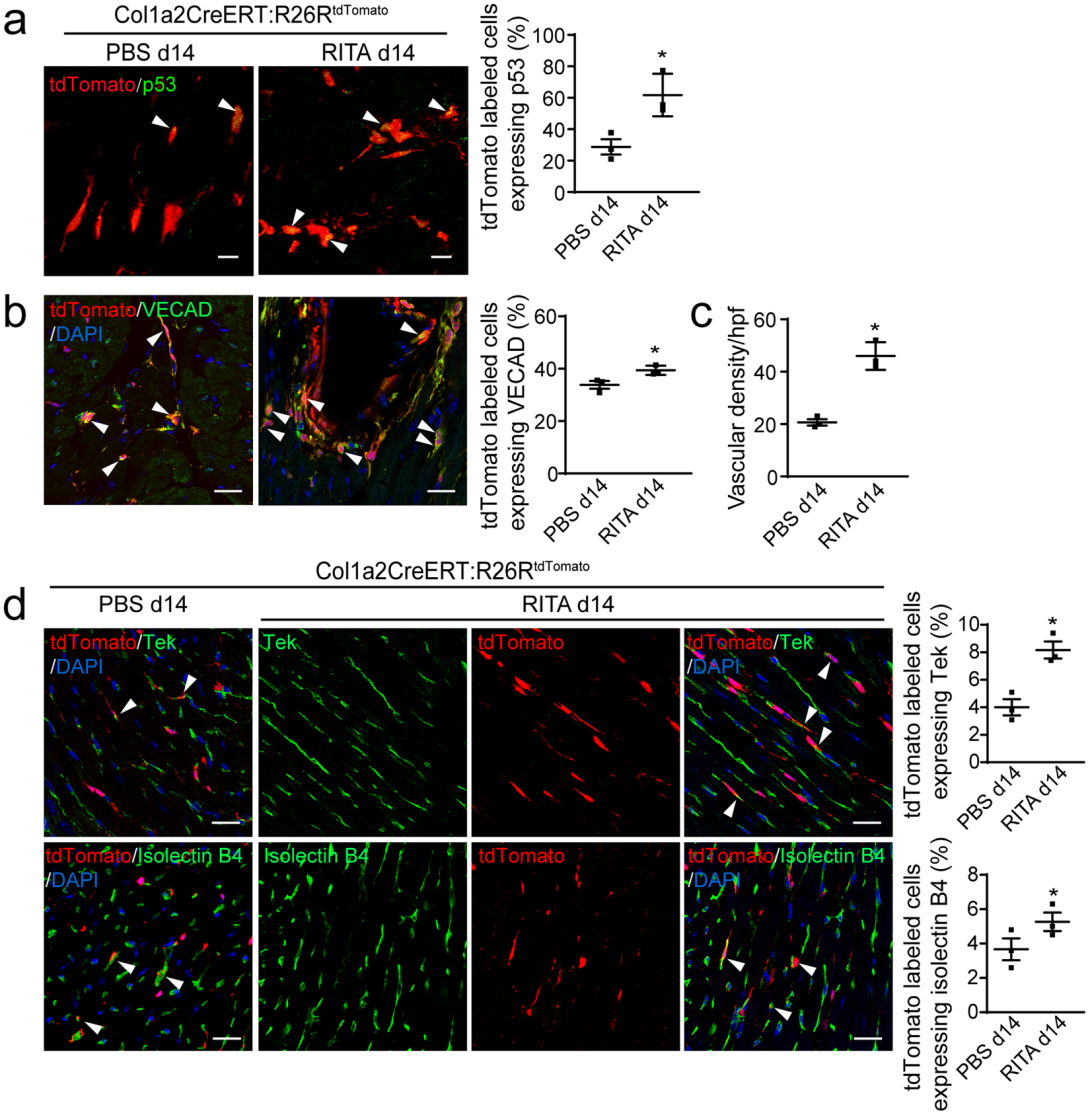
p53 Activator administration (RITA) increase p53 expression and mesenchymal-endothelial transition (MEndoT) after transverse aortic constriction (TAC). Phosphate-buffered saline (PBS) and RITA-treated Col1a2-CreERT: R26R^tdTomato^ mice 14 day post TAC (**a**) Immunostaining of p53 and percentage of tdTomato-labeled cells expressing p53. (**b**) Immunofluorescence staining for endothelial markers vascular endothelial cadherin (VECAD) and (**c**) vascular density per high-power field (hpf) in (**b**). (**d**) Immunofluorescence staining and percentage of tdTomato-labeled cells expressing endothelial markers Tek and isolectin B4. Graphs show mean ± S.E.M.; n = 3 animals/group, *p < 0.05, using an unpaired t-test compared with PBS control after TAC. Colocalization of fluorophores indicated by arrowhead. Scale bar: 25 µm.

We next investigated if stimulation of p53 signaling by administration of RITA (reactivating p53 and inducing tumor apoptosis) enhances MEndoT and protects against cardiac hypertrophy after TAC. We observed that by 14 days after TAC, p53-expressing cells in tdTomato-labeled cardiac fibroblasts doubled after RITA treatment (Fig. 5a). Compared to PBS-treated Col1a2-CreERT: R26R^tdTomato^ mice, tdTomato-labeled cardiac fibroblasts expressing VECAD increased by 17.9% 14 days post-TAC after RITA treatment, leading to a 24% increased vascular density in heart sections (Fig. 5b,c). Moreover, other endothelial markers that were expressed at low levels in MEndoT-derived cells were also more significantly enhanced after RITA treatment. The expression of Tek and isolectin B4 in tdTomato-labeled cardiac fibroblasts increased by 112.5% and 70.9% after RITA treatment, respectively(Fig. 5d).

In TCF21-MerCreMer: R26R^tdTomato^ mice, tdTomato-labeled cardiac fibroblasts expressing VECAD, Tek, and isolectin B4 increased by 57.6%, 76.9%, and 57.6% 14 days post TAC after RITA treatment, respectively (Supplemental Fig. 9a–c). Interestingly, we did not observe significant changes in the expression of CD31 in tdTomato-labeled cells after RITA treatment of Col1a2-CreERT: R26R^tdTomato^ mice. However, the tdTomato-labeled cardiac fibroblasts expressing CD31 were significantly increased in TCF21-MerCreMer: R26R^tdTomato^ mice after RITA treatment (Supplemental Fig. 9d), which indicate that the characteristics of MEndoT-derived cells may be different for their lineage origin.

The improved expression of different endothelial cell markers in MEndoT-derived cells after RITA administration also lead to an increased contribution of MEndoT to corresponding endothelial marker positive cells (Supplemental Fig. 9e). Endothelial markers such as isolectin B4 and CD31 are considered markers for cardiac microvascular endothelial cells. These results suggest that RITA administration modulated MEndoT by a combined effect that contributed to the expression of different endothelial markers to different levels which are finally related to functional maturity of MEndoT-derived cells.

**Figure 6:**
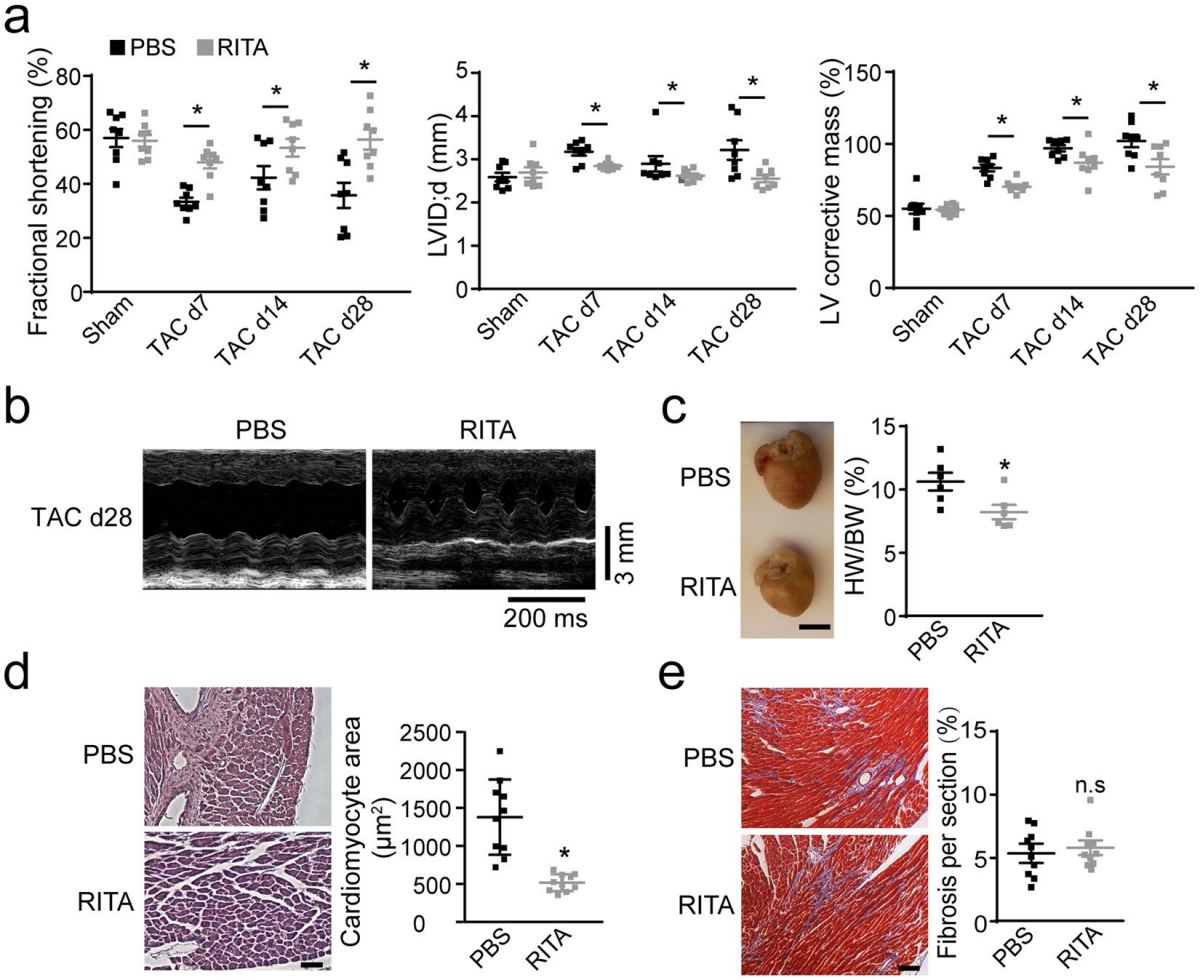
RITA preserves heart function and attenuates cardiac hypertrophy after transverse aortic constriction (TAC). (**a**) Echocardiographic assessment of cardiac function in phosphate-buffered saline (PBS)- or RITA-treated mice prior to and post-TAC (n = 10 animals/group). (**b**) Representative M-mode echocardiograms in (**a**). (**c**) Ratio of heart to body weight in PBS- and RITA-treated mice 5 weeks TAC (n = 6 animals/group). (**d**) Quantitation of cardiomyocyte size in H&E stained heart tissue 5 weeks post-TAC (n = 10 animals/group, Scale bar: 50 µm). (**e**) Quantitation of fibrotic area in Masson’s trichrome stained heart tissue 5 weeks post-TAC (n = 10 animals/group). Graphs show mean ± S.E.M.; *p < 0.05, using ANOVA with post hoc tests, compared to PBS control after TAC. Scale bar: 25 µm.

Echocardiography assay showed significantly attenuated cardiac hypertrophy and preserved heart function in RITA-treated animals compared with the control group (Fig. 6a,b). Furthermore, 5 weeks after TAC, we observed lower heart to body weight ratio and smaller cadiomyocyte size in RITA-treated than PBS-treated mice (Fig. 6c,d). However, Masson’s trichrome staining did not show a significant decrease in interstitial fibrosis in RITA-treated mice (Fig. 6e). These observations suggest that modulation of MEndoT can preserve heart function and attenuate pressure overload-induced cardiac hypertrophy by improving the characteristics of MEndoT-derived cells that may enhance functional angiogenesis after TAC.

**Figure 7:**
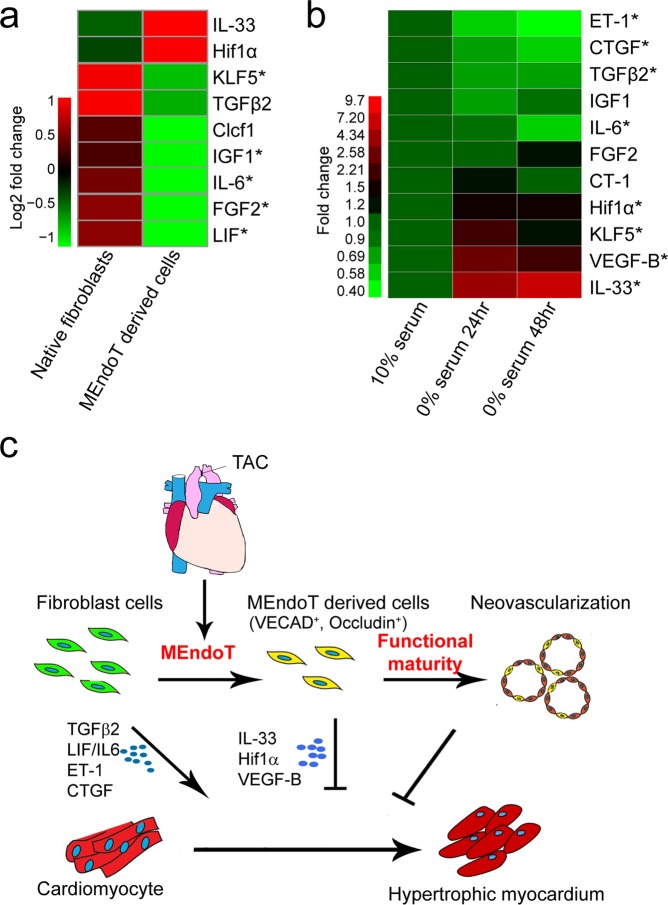
Altered expression of paracrine factors in fibroblasts undergoing mesenchymal-endothelial transition (MEndoT) and a schematic for MEndoT regulation in cardiac hypertrophy. (**a**) RNA sequencing (RNA-seq) assay shows MEndoT-derived cells isolated from hearts of Col1a2-CreERT: R26R^tdTomato^ mice 14 day post-TAC exhibit altered expression of paracrine factors demonstrated to be important for cardiac hypertrophy and heart failure (*q < 0.05, n = 3 animals/group). (**b**) Quantitative polymerase chain reaction (qPCR) analysis of expression of paracrine factors in fibroblasts undergoing MEndoT using *in vitro* serum starvation model. Expression level shown are average fold-change to fibroblasts in 10% serum.(n = 3 experiment repeat, *p < 0.05, using an unpaired t-test as indicated). (**c**) A schematic for MEndoT regulation in cardiac hypertrophy.

During the development of cardiac hypertrophy, a variety of growth factors produced by fibroblasts mediate the interplay between cardiac fibroblasts and cardiomyocytes, such that fibroblasts enhance cardiomyocyte hypertrophy and cardiac cardiomyocytes regulates fibroblast adhesion or proliferation^25–27^. The RNA-seq showed that MEndoT-derived cells upregulated anti-hypertrophic factors such as interleukin (IL)-33^28^ and hypoxia-inducible factor-1, alpha (HIF1α)^29^, and downregulated pro-hypertrophic factors such as transforming growth factor (TGF)-β2^30^, cardiotrophin-like cytokine factor 1 (Clcf1)^31^, IL-6/leukemia inhibitory factor (LIF)^32^, Kruppel-like factor 5 (KLF5)^33^, insulin like growth factor1(IGF1)^34^, and FGF2 (fibroblast growth factor 2)^35^ (Fig. 7a and Supplemental Table 3).

Using an *in vitro* model where fibroblasts undergo MEndoT under serum starvation, we further determined the expression of the known paracrine factors that have been demonstrated to play roles in cardiac hypertrophy using qPCR (Fig. 7b and Supplemental Fig. 10). We observed that fibroblasts undergoing MEndoT *in vitro* showed downregulated expression of most of pro-hypertrophic factors such as endothelin (ET)-1^36^, connective tissue growth factor (CTGF)^37^, TGFβ2, IGF1(regulated by KLF5), IL6, and cardiotrophin (CT)-1^31^, and upregulate anti-hypertrophic factors such as IL33, HIF1α, and vascular endothelial growth factor, B (VEGFB)^38,39^.

## Discussion

Reducing fibrosis and forming new blood vessel is an important mechanism for cardiac repair. In addition to studies of preexisting coronary endothelial cells, a large body of work over the last decade suggests that non-endothelial cell sources such as C-Kit and Sca-1-positive cells can also be a substantial source of endothelial cells^40–42^. Recently, He *et al*. reported that MEndoT does not occur and only the preexisting coronary vessels mediates neovascularization after cardiac injury^43^. He *et al*. investigated MEndoT using multiple strains of transgenic mice, however, only two endothelial cell markers(VECAD and CD31) were investigated^43^. Considering the potential difference of the sources of Col1a2CreERT strain mice, we introduced TCF21MerCreMer transgenic mice. We do observed similar results that MEndoT-derived cells is lack of the expression of CD31. However, we still could not figure out why the expression of VECAD in MEndoT-derived cells were not detected by He *et al*.^43^. A possible cause may be the difference of antibodies and reagents used. Therefore, investigating more endothelial markers is a useful method to avoid the problems due to the antibodies.

The main purpose of current work is to determine the pathophysiological significance of MEndoT in cardiac hypertrophy. Because CMEC plays important role on the function of cardicmyocytes^8,10^, we focused on the genetic expression of endothelial markers of CMEC, which can provide an insight into the mechanism of MEndoT-derived cells in anti-cardiac hypertrophy. Although MEndoT-derived cells exhibited a similar high expression of some markers such as VECAD and occludin as native endothelial cells, they showed a significantly lower expression of most endothelial markers, such as Tek, isolectin B4, and vWF, than native endothelial cells did. The expression of these endothelial marker may vary depending on the cardiac injury model (e.g., eNOS) and origin of fibroblast lineage (e.g., vWF and CD31). Besides, RNA-seq results further show more endothelial cell markers were upregulated in MEndoT-derived cells (Fig. 3 and Supplemental Fig. 5). Thus, our results demonstrate that MEndoT-derived cells may represent a new substantial source of endothelial cells after cardiac injury, and they constitute a highly heterogeneous cell sub-population with greater diversity than native endothelial cells.

Investigation of the genetic expression of different endothelial cell surface markers is essential for understanding the function of MEndoT-derived cells in cardiovascular disease. Among the endothelial markers we detected, one of most widely used endothelial markers CD31 exhibits significantly difference between the native endothelial cell population and MEndoT-derived cells. However, the pathophysiological significance of the very low expression of CD31 in MEndoT-derived cells is unknown. CD31-deficient mice have vasculature and normal baseline endothelium^44^ and CD31-positive cells do not appear to be the main endothelial cells as shown recently by Pinto *et al*.^45^. Our result also show that the expression of CD31 in MEdnoT-derived cells can be improved by administration of RITA. Therefore, how to define the potential phenotypic properties and characteristics of MEndoT-derived cells by different endothelial cell surface markers, is mainly relied on our understanding about the relationship between the expression of endothelial markers and the function of different endothelial cell populations.

Our previous work demonstrated that the main origin of MEndoT-derived cells is from fibroblasts, which showed that a fraction of endothelial cells that expressed VECAD without tdTomato fluorescence and conversely, tdTomato labeled cells from Col1a2-CreERT:R26R^tdTomato^ mice expressed endothelial markers but not fibroblast marker collagen1^3^. These observations indicate that it is unlikely that endothelial cells expressing collagen1 recombined and substantially contributed to the population of tdTomato^+^ cells expressing endothelial markers^3^. However, how much characteristics of fibroblast were kept in MEndoT-derive cells is still lack of in-depth research. Therefore, to better define the highly heterogeneous MEndoT-derive cells, besides the expression characteristics of different cell markers, functional assay combined with some new technology such as single cell RNA-seq will be considered in the future work.

The results from mice with p53 specifically knocked down in cardiac fibroblasts demonstrate the MEndoT is p53 dependent. Our previous work has also confirmed the strong correlation between p53 and MEndoT^3^. However, p53 is a key master regulator and stimulating p53 signal pathway by RITA may also have effect on other cell types. Literature demonstrated that p53 does not play a role in mediating cardiomyocyte apoptosis after cardiac injury^46^ and our previous work also did not find administration of RITA was associated with increased TUNEL staining in cardiomyocyte^3^. Furthermore, the previous literature showed that proliferation of preexisted endothelial cells only benefit from the deletion of p53 after injury^29^. Therefore, eventhough we could not exclude potential unknown effects of RITA on other cardiac cells that could potentially influence heart function, we focused on the effects of RITA on MEndoT, which is the central phenomenon discussed in this manuscript. Because our previous work have demonstrated that MEndoT can contribute to functional vasculature^3^. Thus, finding the new possible target genes that can specifically regulating MEndoT and improve the functional maturity of MEndoT-derived cells will be our future direction.

In addition, we observed that regulation of MEndoT-derived cells also altered the expression of paracrine factors by fibroblasts, which may directly affect cardiomyocytes and attenuate cardiac hypertrophy. These observations suggest that the alteration of paracrine factors in MEndoT-derived cells is another important mechanism by which MEndoT regulation attenuates cardiac hypertrophy and preserves heart function. Therefore, MEndoT-derived cells could be a regulatory target for cardiac hypertrophy, which contributes to cardiac repair not only by improving neovascularization but also altering the paracrine effect of fibroblasts *in vivo* (Fig. 7c). Identifying the phenotypic properties and characteristics of MEndoT-derived cells, and finding new mechanism of how MEndoT-derived cells were precisely regulated and transformed into specific functional endothelial cell population will provide us a new strategy for the treatment of cardiac hypertrophy in the future.

## Materials and Methods

### Mice

All animal studies were approved by the Institutional Animal Care and Use Committee of the Guangzhou Medical University(the acceptance number:2016–025) and all animal procedures conform to the National Institutes of Health (NIH) guidelines. The TCF21-MerCreMer mice were introduced from UT Southwestern^18^. The Tek-CreERT mice and Tie2GFP mice were bought from the Jackson Lab. All mice were of a C57BL/6 background, and the Col1a2-CreERT: R26R^tdTomato^ and Col1a2-CreERT: R26R^tdTomato^: p53CKO mice used were the same strain used previously^3^. Tamoxifen (1 mg, Sigma-Aldrich, St. Louis, MO, USA) was injected intraperitoneally for 10 days to induce Cre-mediated recombination in Col1a2-CreERT mice. Five days after cessation of tamoxifen the animals were subjected to injury and 24 h later, the Reactivating p53 and inducing tumor apoptosis (RITA)-treated mice were administered RITA (Millipore, Billerica, MA, USA) intraperitoneally at 0.3 mg·kg^−1^·day^−1^ for 5 days per week until harvest.

### Murine cardiac hypertrophy model

We randomly allocated 8–9-week-old mice to sham or TAC injury groups and the investigators performing the surgeries and cardiac function studies were blinded to the mouse genotype and treatment. TAC was performed as follows. Each mouse was anesthetized using 2.0% isoflurane, placed on a heated surgical board, and intubated with a 22-gauge (PE90) plastic catheter. The catheter was connected to a volume-cycled ventilator supplying supplemental oxygen at a tide volume of 225–250 mL and respiratory rate of 120–130 strokes/min. Surgical plane anesthesia was subsequently maintained with 1.5% isoflurane. A left thoracotomy was performed: the skin was incised, the chest cavity was opened at the level of the second intercostal space, and the transverse section of the aorta was isolated. Transverse aortic constriction was created via a left thoracotomy by placing an Ethicon 7–0 nylon ligature securely around the trans-aorta and a 27-gauge needle, completely occluding the aorta. The needle was removed, restoring the lumen with severe stenosis. The lungs were reinflated, the chest was closed using a Vicryl 6–0 suture, and the muscle and skin were sutured using a Vicryl 6–0 suture in a running subcuticular pattern. Once the mouse resumed breathing on its own, it was removed from the ventilator and allowed to recover in a clean cage on a heated pad. Sham injury was similarly performed without binding. Ang II-induced cardiac hypertrophy was established by implanting Ang-II infusion osmotic mini-pumps subcutaneously in 8-week-old mice at 1 mg·kg^−1^·day^−1^ for 21 days.

### Echocardiography

The echocardiography was conducted using a VisualSonics Vevo 2100 machine. Conscious echocardiography was performed without anesthesia at baseline and serially following recovery from surgical procedures to track cardiac function. For echocardiography, the hair was removed from the chest of each mouse using NAIR and then it was gently restrained and placed on a platform followed by rapid echocardiography (<5 min). For the conscious echocardiography, the mouse was picked up by the back of the neck and held in one hand with the tail held between the last two fingers. A soft thin rope with an attached rubber protector was then placed around each limb and the animal was restrained on a platform by attaching the end of the rope not attached to the animal to the base of the platform. In our experience, this process is well tolerated by animals. Distress was minimized by performing the echocardiography rapidly. Parasternal long axis M-mode images were recorded, and measurements and analysis were then performed as described. The echocardiographer was blinded to the genotype and treatment of the animal being examined.

### Immunofluorescence, confocal imaging, and quantitation

Mice were anaesthetized by using 2.0% isoflurane after connecting to a ventilator. After the left ventricle was perfused with phosphate-buffered saline (PBS) and paraformaldehyde (PFA), the heart was further fixed for 4 h in 2% PFA at 4 °C, followed by cryoprotection by immersion in 30% sucrose solution overnight before freezing in optimal cutting temperature (OCT) solution. Immunofluorescent staining of frozen sections (7 µm) was performed using primary antibodies to vascular endothelial cadherin (VECAD, ab33168), CD31 (ab28364), von Willebrand factor (vWF, ab11713), endothelial nitric oxide synthase (eNOS, ab5589), occludin (ab31721, all from Abcam, Cambridge, UK), Tek (AF762, R&D Systems, Minnesota, MN, USA), isolectin B4 (B-1205, Vector Labs, Burlingame, CA, USA), p53 (ab31333, Abcam), and associated fluorescein-conjugated secondary antibodies following the manufacturer instructions. Labelled sections were probed, and images were captured using a Leica TCS SP8 confocal microscope. Then, 8–10 fields were randomly chosen from each section of the heart, images were captured at ×63 magnification using a confocal microscope, and were each defined as one area. Then, co-localization analysis of confocal images was performed using the ImageJ software (National Institutes of Health [NIH]). Masson’s trichrome staining was performed on heart sections as described previously.

### Fibroblast isolation and culture

Uninjured mice were euthanized by using 2.0% isoflurane after connecting to a ventilator and cardiac fibroblasts were isolated from the hearts as previously described. Briefly, 8-week-old adult mouse hearts were excised, minced, and sequentially digested in a solution containing 50 U/mL collagenase II and 0.1% trypsin at 37 °C. The cells were collected and seeded in culture dishes for 90 min to allow preferential attachment of fibroblasts, after which unattached cells were rinsed off. The cells were maintained in Iscove’s modified Dulbecco’s medium (IMDM) supplemented with penicillin/streptomycin, 10% fetal bovine serum (FBS), and 10 ng/mL each of leukemia inhibitory factor (LIF) and 10 ng/m fibroblast growth factor (FGF) until they became confluent within 7–10 days. Serum-starved or unstarved cells were cultured in IMDM plus penicillin/streptomycin or IMDM plus penicillin/streptomycin and 10% FBS, respectively. The cell culture medium for serum starvation was replaced daily.

### Quantitative real-time polymerase chain reaction (PCR)

RNA was isolated from cardiac fibroblasts and reverse transcribed using the SV Total RNA Isolation System (Promega, Madison, WI, USA) and the Reverse Transcription System (Promega, Madison, WI, USA). Quantitative polymerase chain reaction (qPCR) was performed using the 2× RealStar Green Fast Mixture (GenStar, Beijing, China) using an CFX96 thermal cycler (Bio-Rad, Burlingame, CA, USA). Fold-changes in gene expression were calculated using the 2^(−△△Ct)^ method after normalizing to glyceraldehyde 3-phosphate dehydrogenase (GAPDH).

### Flow cytometry

Flow cytometric analysis of cell surface markers was performed using CD31-Brilliant Violet 421 (102423, Biolegend, San Diego, CA, USA) and isolectin B4 (B-1205, Vector Labs) antibodies. For isolectin B4, cell fixation and permeability were performed following the protocol of the BD flow kit and DyLight 488 Streptavidin was used as the secondary antibody (SA-5488, Vector Lab). Non-myocytes were isolated from the heart using the fibroblast isolation protocol but the cells were seeded in fibronectin-coated culture dishes overnight. After 24 h minimum culture, the non-myocytes were dissociated using Accutase (Millipore, Billerica, MA, USA) and immunostained with flow cytometric staining buffer (1% FBS + 0.1% NaN_3_ in PBS) for 30 min at 4 °C, followed by washing with the washing buffer and subsequent analysis using a Beckman-Coulter (Dako) CytoFLEX S. The data obtained were analyzed and presented using Flowjo software. The negative control group was processed without primary antibody first and subjected to all other processes the experimental groups were exposed to. Flow cytometric sorting for RNA-sequencing (RNA-seq) was immediately performed after isolating non-myocytes using the BD Influx (BD Biosciences, CA, USA).

### RNA-Seq

The amplification of cDNA were synthesized by using the Discover-sc WTA Kit V2 (Vazyme, N711). Briefly, after a special adapter was added to the 3’ end of the first-strand cDNA, the cDNA was amplified by PCR and the aimed products were purified finally by VAHTS DNA Clean Beads (Vazyme, N411). cDNA concentration and the fragment size were measured by Qubit DNA Assay Kit in Qubit 3.0 Flurometer (Life Technologies, CA, USA) and the Agilent Bioanalyzer 2100 system (Agilent Technologies, CA, USA), respectively.

For each sample, 1 ng qualified WTA of cDNA was used to generate the library following the manufacturer’s recommendations of the TruePrep DNA Library Prep Kit V2 (Vazyme, TD503). In brief, sequencing adapter was added to the 3’ adenosine on the randomly fragmented cDNA followed by PCR. Then, after purification with VAHTS DNA Clean Beads, the concentration of library was measured by using Qubit DNA Assay Kit. Insert size of the generated library was assessed by the Agilent Bioanalyzer 2100 system and qualified further accurately by Step One Plus Real-Time PCR system (ABI, USA).

After clustering on a cBot Cluster Generation System (Illumina), the prepared library were sequenced on an Illumina Hiseq X Ten platform with 150 bp paired-end module. After quality control, the index of reference genome was obtained by Bowtie2 (v2.2.9)^47^ and the paired-end clean reads were aligned to the reference genome using TopHat (v2.1.1)^48^. Then the mapped reads were assembled by using Cufflinks (v2.2.1)^49^ with a reference-based approach. The Fragments per kilobase of exon per million reads mapped (FPKMs) were calculated by Cuffdiff (v1.3.0) and the differential expression were analyzed by Cuffdiff (v2.2.1)^49^. The genes with corrected p values less than 0.05 were assigned as significant difference.

GO enrichment analysis of differentially expressed genes was implemented with perl module (GO::TermFinder)^24^ and the KEGG pathways were analysed in KEGG database developed by Kanehisa Laboratories^23^. For Go enrichment and KEGG pathways, if the corrected p-value less than 0.05, it will be considered to be significantly enriched among the differentially expressed genes.

### Statistical analysis

Statistical analysis was performed using the GraphPad software (Prism) using the Student’s *t*-test (two-tailed), one- or two-way analysis of variance (ANOVA) with Bonferroni post-hoc test analysis as appropriate. A *p* = 0.05 was considered statistically significant and the mean ± standard error of the mean (S.E.M.) are presented graphically. All results in the text are also shown as mean ± S.E.M.

## Supplementary information

Supplementary Information.

## References

[1] Hunter JJ, Chien KR (1999). Signaling Pathways for Cardiac Hypertrophy and Failure. N. Engl. J. Med..

[2] Nag AC (1980). Study of non-muscle cells of the adult mammalian heart: a fine structural analysis and distribution. Cytobios.

[3] Ubil E (2014). Mesenchymal-endothelial transition contributes to cardiac neovascularization. Nature.

[4] Brumm AJ (2017). Astrocytes Can Adopt Endothelial Cell Fates in a p53-Dependent Manner. Mol. Neurobiol..

[5] Moore, J. B. *et al*. The Epigenetic Regulator HDAC1 Modulates Transcription of a Core Cardiogenic Program in Human Cardiac Mesenchymal Stromal Cells Through a p53-Dependent Mechanism. *Stem Cells Dayt. Ohio*, 10.1002/stem.2471 (2016). 10.1002/stem.2471 PMC5123951 27501845

[6] Cecilia G, Elisabetta D (1997). Heterogeneity of Endothelial Cells. Arterioscler. Thromb. Vasc. Biol..

[7] Kumar S, West DC, Ager A (2010). Heterogeneity in endothelial cells from large vessels and microvessels. Differentiation.

[8] Ando H, Kubin T, Schaper W, Schaper J (1999). Cardiac microvascular endothelial cells express α-smooth muscle actin and show low NOS III activity. Am. J. Physiol. Heart Circ. Physiol..

[9] Ismail JA, Poppa V, Kemper LE, Scatena M, Murry CE (2003). Immunohistologic labeling of murine endothelium. Cardiovasc. Pathol..

[10] Li JM, Adrian MM, Shah AM (2001). Phenotypic Properties and Characteristics of Superoxide Production by Mouse Coronary Microvascular Endothelial Cells. J. Mol. Cell. Cardiol..

[11] Gräfe M, Auch-Schwelk W, Graf K, Terbeek D, Fleck E (1995). Isolation and characterization of macrovascular and microvascular endothelial cells from human hearts. Am. J. Physiol..

[12] Talavera-Adame D, Ng TT, Gupta A, Kurtovic S, Dafoe DC (2011). Characterization of microvascular endothelial cells isolated from the dermis of adult mouse tails. Microvasc. Res..

[13] Marelli-Berg FM, Peek E, Lidington EA, Stauss HJ, Lechler RI (2000). Isolation of endothelial cells from murine tissue. J. Immunol. Methods.

[14] Cha ST, Talavera D, Demir E, Nath AK, Sierra-Honigmann MR (2005). A method of isolation and culture of microvascular endothelial cells from mouse skin. Microvasc. Res..

[15] Duan J (2012). Wnt1/βcatenin injury response activates the epicardium and cardiac fibroblasts to promote cardiac repair. EMBO J..

[16] Kapoor M (2008). GSK-3beta in mouse fibroblasts controls wound healing and fibrosis through an endothelin-1-dependent mechanism. J. Clin. Invest..

[17] Zheng B, Zhang Z, Black CM, de Crombrugghe B, Denton CP (2002). Ligand-Dependent Genetic Recombination in Fibroblasts. Am. J. Pathol..

[18] Acharya A, Baek ST, Banfi S, Eskiocak B, Tallquist MD (2011). Efficient inducible Cre-mediated recombination in Tcf21cell lineages in the heart and kidney. genesis.

[19] Acharya A (2012). The bHLH transcription factor Tcf21 is required for lineage-specific EMT of cardiac fibroblast progenitors. Development.

[20] Pillai ICL (2017). Cardiac Fibroblasts Adopt Osteogenic Fates and Can Be Targeted to Attenuate Pathological Heart Calcification. Cell Stem Cell.

[21] Forde A, Constien R, Gröne H-J, Hämmerling G, Arnold B (2002). Temporal Cre-mediated recombination exclusively in endothelial cells using Tie2 regulatory elements: Characterization of Tamoxifen-Inducible Tie2 Cre Mouse Line. genesis.

[22] Kisanuki YY (2001). Tie2-Cre Transgenic Mice: A New Model for Endothelial Cell-Lineage Analysis *in Vivo*. Dev. Biol..

[23] Minoru K, Susumu G (2000). KEGG: Kyoto Encyclopedia of Genes and Genomes. Nucleic Acids Res..

[24] Boyle EI (2004). GO::TermFinder—open source software for accessing Gene Ontology information and finding significantly enriched Gene Ontology terms associated with a list of genes. Bioinformatics.

[25] Takeda N, Manabe I (2011). Cellular Interplay between Cardiomyocytes and Nonmyocytes in Cardiac Remodeling. Int. J. Inflamm..

[26] Fujiu K, Nagai R (2014). Fibroblast-mediated pathways in cardiac hypertrophy. J. Mol. Cell. Cardiol..

[27] Kakkar R, Lee RT (2010). Intramyocardial fibroblast myocyte communication. Circ. Res..

[28] Sanada S (2007). IL-33 and ST2 comprise a critical biomechanically induced and cardioprotective signaling system. J. Clin. Invest..

[29] Sano M (2007). p53-induced inhibition of Hif-1 causes cardiac dysfunction during pressure overload. Nature.

[30] Dobaczewski M, Chen W, Frangogiannis NG (2011). Transforming growth factor (TGF)-β signaling in cardiac remodeling. J. Mol. Cell. Cardiol..

[31] Kuwahara K (1999). Involvement of cardiotrophin-1 in cardiac myocyte-nonmyocyte interactions during hypertrophy of rat cardiac myocytes *in vitro*. Circulation.

[32] King KL (1998). Phenylephrine, endothelin, prostaglandin F2alpha’ and leukemia inhibitory factor induce different cardiac hypertrophy phenotypes *in vitro*. Endocrine.

[33] Takeda N (2010). Cardiac fibroblasts are essential for the adaptive response of the murine heart to pressure overload. J. Clin. Invest..

[34] McMullen JR (2008). Role of insulin-like growth factor 1 and phosphoinositide 3-kinase in a setting of heart disease. Clin. Exp. Pharmacol. Physiol..

[35] Schultz JE (1999). Fibroblast growth factor-2 mediates pressure-induced hypertrophic response. J. Clin. Invest..

[36] Harada M (1997). Significance of ventricular myocytes and nonmyocytes interaction during cardiocyte hypertrophy: evidence for endothelin-1 as a paracrine hypertrophic factor from cardiac nonmyocytes. Circulation.

[37] Hayata N (2008). Connective tissue growth factor induces cardiac hypertrophy through Akt signaling. Biochem. Biophys. Res. Commun..

[38] Huusko J (2012). AAV9-mediated VEGF-B gene transfer improves systolic function in progressive left ventricular hypertrophy. Mol. Ther. J. Am. Soc. Gene Ther..

[39] Xu XH (2011). VEGF attenuates development from cardiac hypertrophy to heart failure after aortic stenosis through mitochondrial mediated apoptosis and cardiomyocyte proliferation. J. Cardiothorac. Surg..

[40] van Berlo JH (2014). c-kit+ cells minimally contribute cardiomyocytes to the heart. Nature.

[41] Uchida S (2013). Sca1-Derived Cells Are a Source of Myocardial Renewal in the Murine Adult Heart. Stem. Cell Rep..

[42] Jackson KA (2001). Regeneration of ischemic cardiac muscle and vascular endothelium by adult stem cells. J. Clin. Invest..

[43] He L (2017). Preexisting endothelial cells mediate cardiac neovascularization after injury. J. Clin. Invest..

[44] Goel R (2007). The proinflammatory phenotype of PECAM-1-deficient mice results in atherogenic diet-induced steatohepatitis. Am. J. Physiol. Gastrointest. Liver Physiol..

[45] Pinto AR (2016). Revisiting Cardiac Cellular Composition. Circ. Res..

[46] Bishopric NH (1999). Hypoxia-activated apoptosis of cardiac myocytes requires reoxygenation or a pH shift and is independent of p53. J. Clin. Invest..

[47] Langmead B, Salzberg SL (2012). Fast gapped-read alignment with Bowtie 2. Nat. METHODS.

[48] Daehwan (2013). TopHat2: accurate alignment of transcriptomes in the presence of insertions, deletions and gene fusions. Genome Biol..

[49] Trapnell C (2012). Differential gene and transcript expression analysis of RNA-seq experiments with TopHat and Cufflinks. Nat. Protoc..

