# Supplementary material for: Mesenchymal-endothelial transition-derived cells as a potential new regulatory target for cardiac hypertrophy
Source: Sci Rep. 2020 Apr 20;10:6652. doi: 10.1038/s41598-020-63671-8 (PMC7170918; doi:10.1038/s41598-020-63671-8)
Supplement: Supplementary file 1 — Supplementary Information. [file 41598_2020_63671_MOESM1_ESM.pdf]

Supplemental Materials

Mesenchymal-endothelial transition-derived cells as a potential new regulatory target for cardiac hypertrophy

Wenyan Dong[1*] , Ruiqi Li[1*] , Haili Yang[1], Yan Lu[2], Longhai Zhou[1], Lei Sun[1], Dianliang Wang[3#], and Jinzhu Duan[1#]

[1] Heart Center and Institute of Pediatrics, Guangzhou Women and Children's Medical Center, Guangzhou Medical University, Guangzhou 510120, China

[2] Department of Pathology, University of Washington, Seattle 98109, WA, USA

[3] Stem Cell and Tissue Engineering Research Laboratory, PLA Rocket Force Characteristic Medical Center, Beijing 100088, China

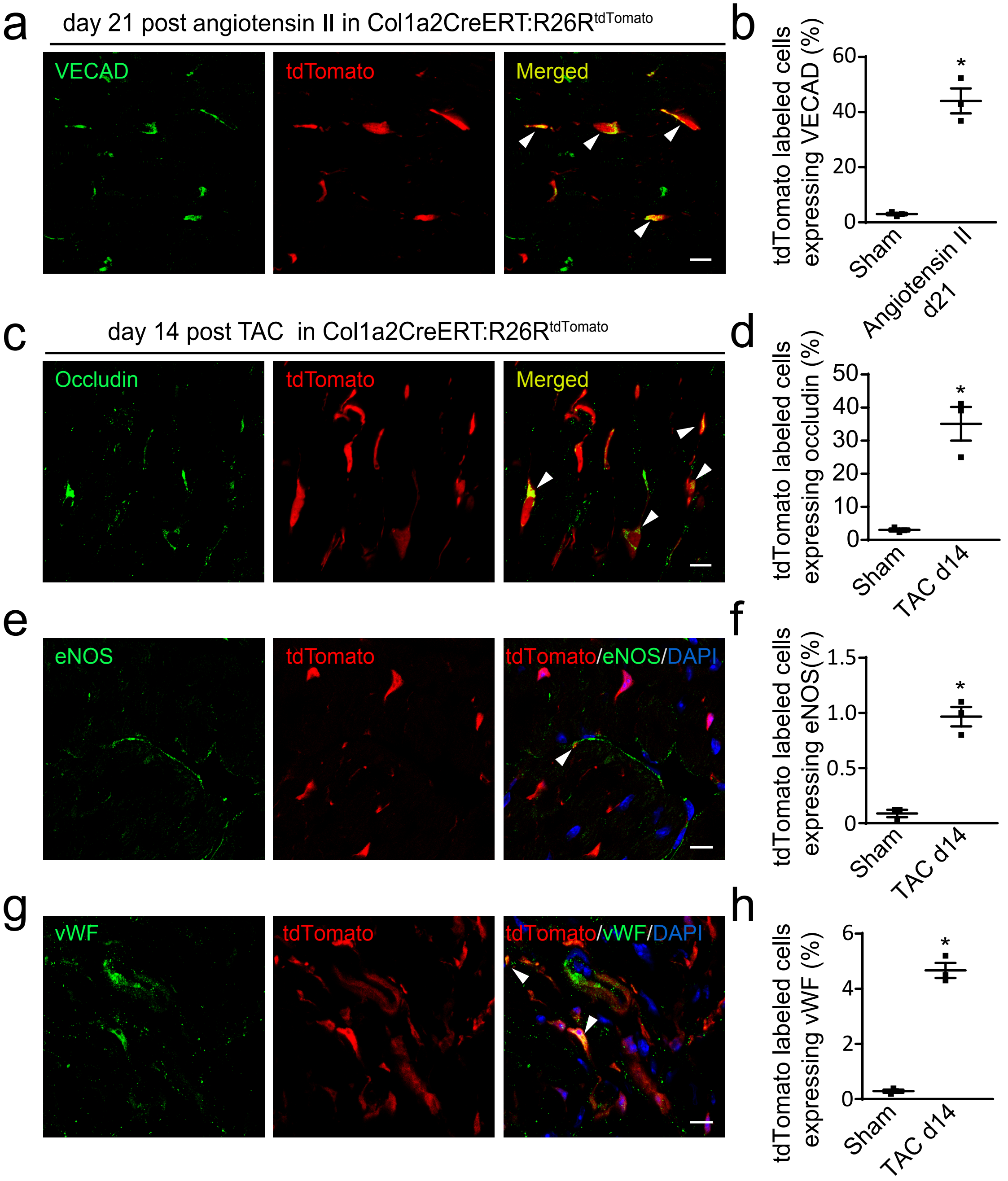
a
day 21 post angiotensin II in Col1a2CreERT:R26R tdTomato
VECAD
tdTomato
Merged
b
tdTomato labeled cells expressing VECAD (%)
60
40
20
0
Sham
Angiotensin II d21
*
c
day 14 post TAC in Col1a2CreERT:R26R tdTomato
Occludin
tdTomato
Merged
d
tdTomato labeled cells expressing occludin (%)
50
40
30
20
10
0
Sham
TAC d14
*
e
eNOS
tdTomato
tdTomato/eNOS/DAPI
f
tdTomato labeled cells expressing eNOS(%)
1.5
1.0
0.5
0
Sham
TAC d14
*
g
vWF
tdTomato
tdTomato/vWF/DAPI
h
tdTomato labeled cells expressing vWF (%)
6
4
2
0
Sham
TAC d14
*

Supplemental Figure 1: MEndoT in angiotensin II or TAC induced cardiac hypertrophy
(a) Immunofluorescence staining for the endothelial marker VECAD in the heart of Col1a2-CreERT: R26R^{tdTomato} mice 21 days post angiotensin II-induced cardiac hypertrophy and (b) the percentage of labeled cardiac fibroblasts expressing the endothelial marker VECAD in (a).
(c-h) Immunofluorescence staining for endothelial markers in the heart of Col1a2-CreERT: R26R^{tdTomato} mice 14 days post TAC (c) Immunofluorescence staining of occludin and (d) the percentage of labeled cardiac fibroblasts expressing the endothelial marker occludin in (c). (e) Immunofluorescence staining of the endothelial marker eNOS and (f) the percentage of labeled cardiac fibroblasts expressing eNOS in (e). (g) Immunofluorescence staining of endothelial marker vWF and (h) the percentage of labeled cardiac fibroblasts expressing vWF in (g). All graphs show mean±S.E.M. n=3 animals, *$p<0.05$ using an unpaired t-test compared with sham. Colocalization of fluorophores is indicated by the arrowhead. Scale bar: 10 μm.

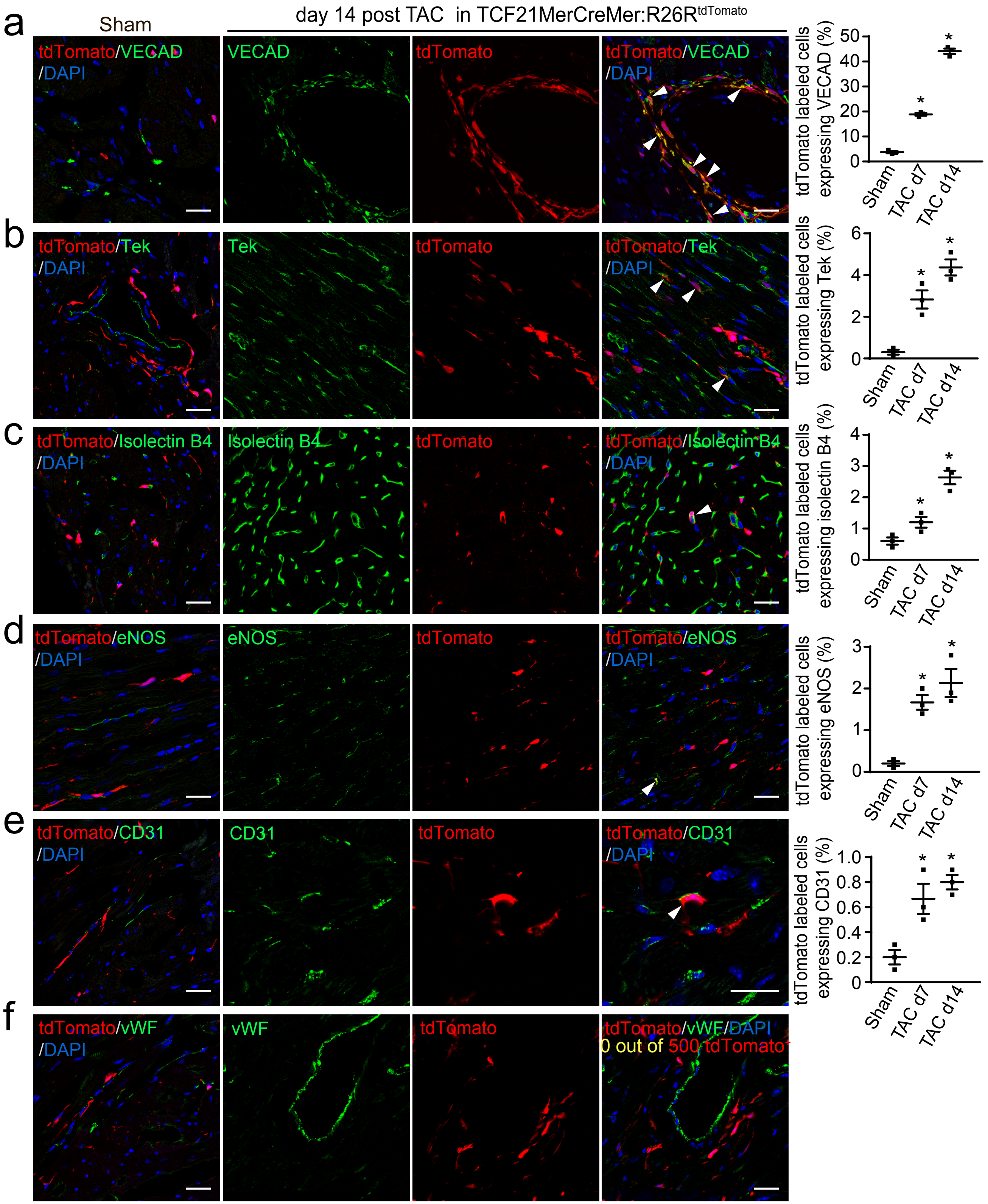
a
Sham
day 14 post TAC in TCF21MerCreMer:R26R tdTomato
tdTomato/VECAD/DAPI
VECAD
tdTomato
tdTomato/VECAD/DAPI
tdTomato labeled cells expressing VECAD (%)
Sham
TAC d7
TAC d14
b
tdTomato/Tek/DAPI
Tek
tdTomato
tdTomato/Tek/DAPI
tdTomato labeled cells expressing Tek (%)
c
tdTomato/Isolectin B4/DAPI
Isolectin B4
tdTomato
tdTomato/Isolectin B4/DAPI
tdTomato labeled cells expressing isolectin B4 (%)
d
tdTomato/eNOS/DAPI
eNOS
tdTomato
tdTomato/eNOS/DAPI
tdTomato labeled cells expressing eNOS (%)
e
tdTomato/CD31/DAPI
CD31
tdTomato
tdTomato/CD31/DAPI
tdTomato labeled cells expressing CD31 (%)
f
tdTomato/vWF/DAPI
vWF
tdTomato
tdTomato/vWF/DAPI
0 out of 500 tdTomato+

Supplemental Figure 2: Immunofluorescence staining of endothelial cell markers post TAC in TCF21-MerCreMer: R26R^{tdTomato} mice

All graphs show mean±S.E.M. n=3 animals, *$p<0.05$ using an unpaired t-test compared with sham. Colocalization of fluorophores is indicated by the arrowhead. Scale bar: 25 µm.

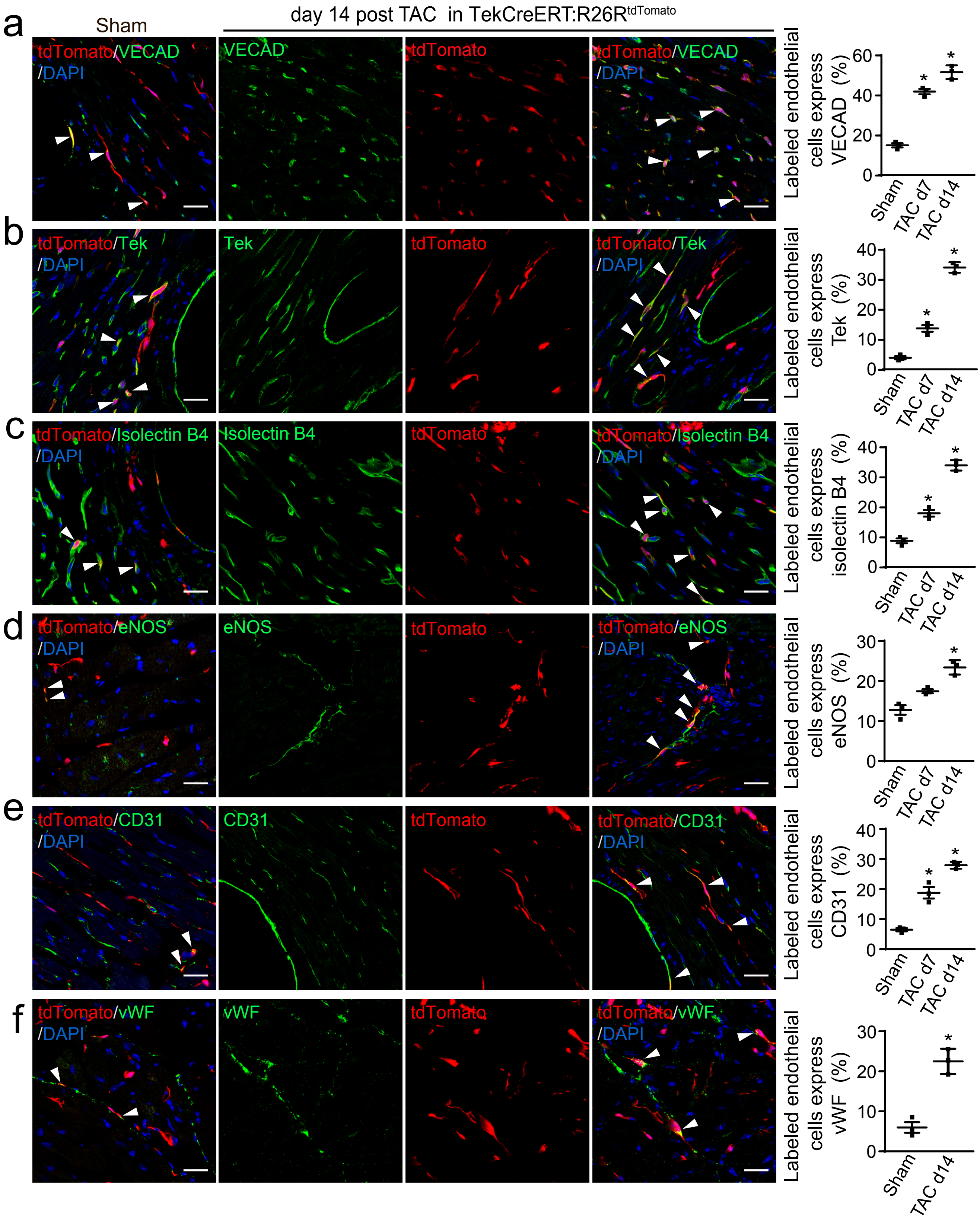
a
b
c
d
e
f
Sham
day 14 post TAC in TekCreERT:R26R^tdTomato
tdTomato/VECAD/DAPI
VECAD
tdTomato
Labeled endothelial cells express VECAD (%)
tdTomato/Tek/DAPI
Tek
Labeled endothelial cells express Tek (%)
tdTomato/Isolectin B4/DAPI
Isolectin B4
Labeled endothelial cells express isolectin B4 (%)
tdTomato/eNOS/DAPI
eNOS
Labeled endothelial cells express eNOS (%)
tdTomato/CD31/DAPI
CD31
Labeled endothelial cells express CD31 (%)
tdTomato/vWF/DAPI
vWF
Labeled endothelial cells express vWF (%)
Sham
TAC d7
TAC d14
*

Supplemental Figure 3: Immunofluorescence staining of endothelial cell markers post TAC in Tek-CreERT: R26R^{tdTomato} mice

All graphs show mean±S.E.M. n=3 animals, *p<0.05 using an unpaired t-test compared with sham. Colocalization of fluorophores is indicated by the arrowhead. Scale bar: 25 μm.

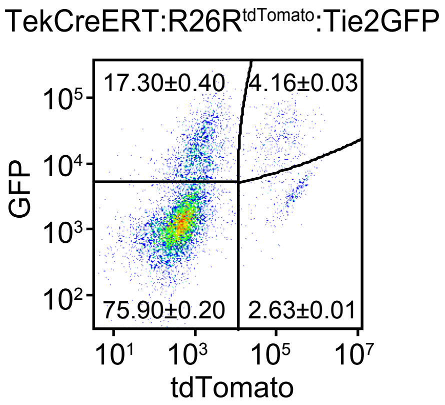

Supplemental Figure 4: Flow cytometry assay for non-myocyte within 24 hours after isolation from Tek-CreERT:R26R^{tdTomato}:Tie2GFP mice

All graphs show mean±S.E.M; n=3 animals.

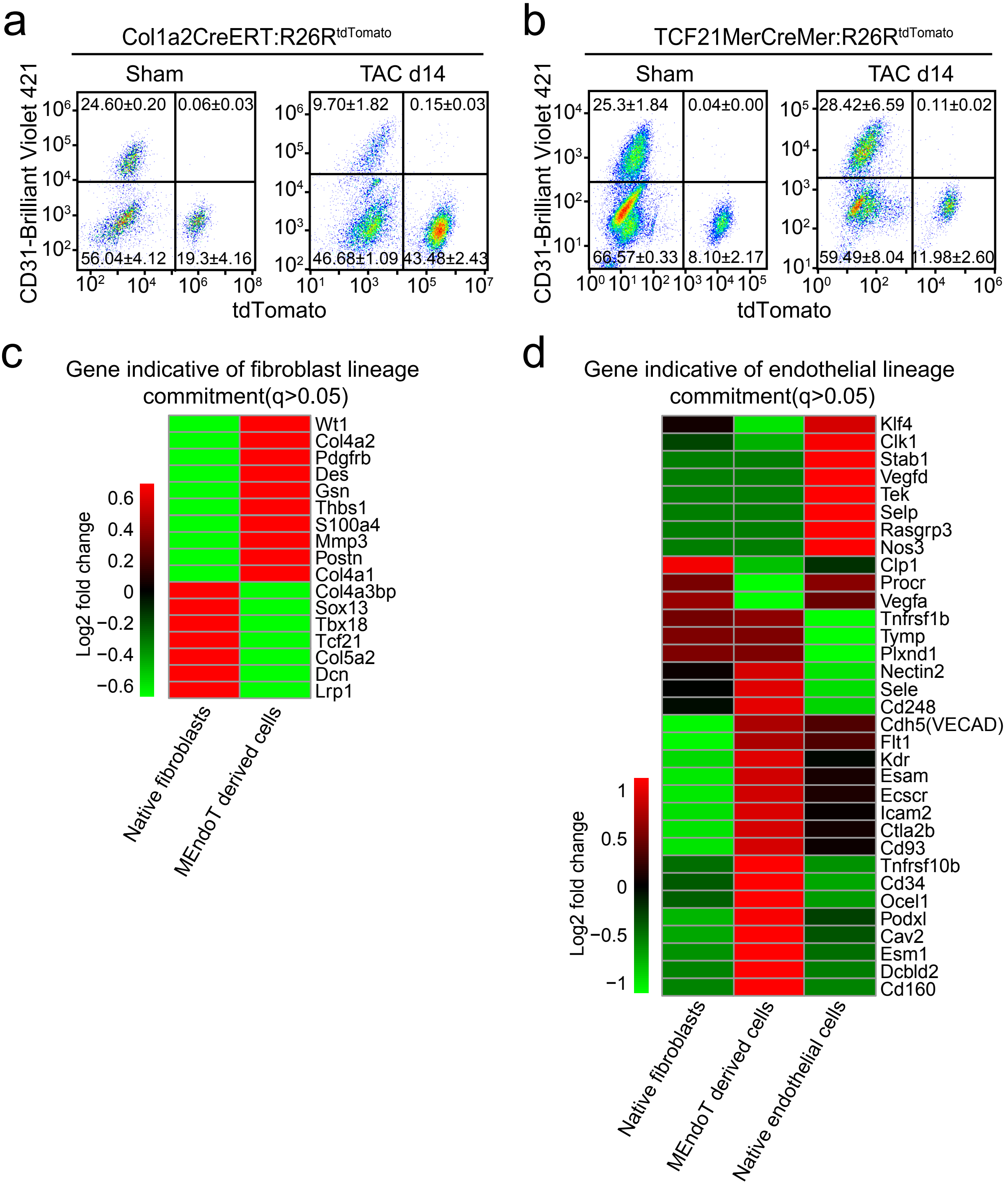
a
Col1a2CreERT:R26R tdTomato
Sham
TAC d14
24.60±0.20
0.06±0.03
56.04±4.12
19.3±4.16
9.70±1.82
0.15±0.03
46.68±1.09
43.48±2.43
CD31-Brilliant Violet 421
tdTomato
b
TCF21MerCreMer:R26R tdTomato
Sham
TAC d14
25.3±1.84
0.04±0.00
66.57±0.33
8.10±2.17
28.42±6.59
0.11±0.02
59.49±8.04
11.98±2.60
CD31-Brilliant Violet 421
tdTomato
c
Gene indicative of fibroblast lineage commitment(q>0.05)
Wt1
Col4a2
Pdgfrb
Des
Gsn
Thbs1
S100a4
Mmp3
Postn
Col4a1
Col4a3bp
Sox13
Tbx18
Tcf21
Col5a2
Dcn
Lrp1
Log2 fold change
Native fibroblasts
MEndoT derived cells
d
Gene indicative of endothelial lineage commitment(q>0.05)
Klf4
Clk1
Stab1
Vegfd
Tek
Selp
Rasgrp3
Nos3
Clp1
Procr
Vegfa
Tnfrsf1b
Tymp
Plxnd1
Nectin2
Sele
Cd248
Cdh5(VECAD)
Flt1
Kdr
Esam
Ecscr
Icam2
Ctla2b
Cd93
Tnfrsf10b
Cd34
Ocel1
Podxl
Cav2
Esm1
Dcbld2
Cd160
Log2 fold change
Native fibroblasts
MEndoT derived cells
Native endothelial cells

Supplemental Figure 5: Heatmap of the fibroblast or endothelial marker genes which were not significantly expressed in MEndoT-derived cells by RNA-seq Assay

(a-b) Flow cytometry assay of CD31 14 days post TAC in Col1a2-CreERT: R26R^{tdTomato} mice and TCF21-MerCreMer:R26R^{tdTomato} mice (mean±S.E.M.; n=3 animals). (c-d) Heatmap of fibroblast and endothelial marker genes that are not statistically significantly expressed in MEndoT-derived cells in comparison with native fibroblasts determined by the RNA-seq assay, as shown in Figure 3.

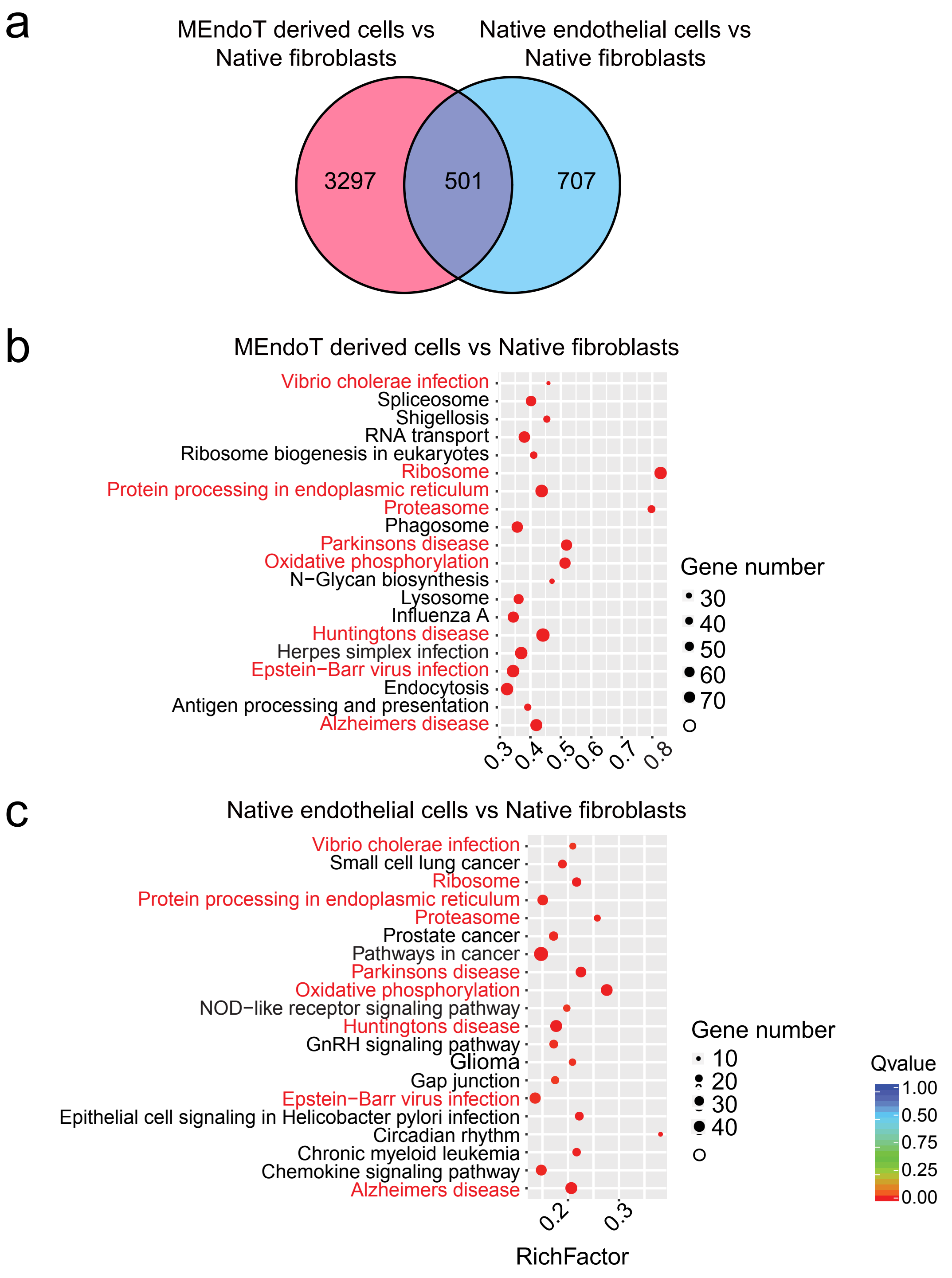
a
MEndoT derived cells vs Native fibroblasts
Native endothelial cells vs Native fibroblasts
3297
501
707
b
MEndoT derived cells vs Native fibroblasts
Vibrio cholerae infection
Spliceosome
Shigellosis
RNA transport
Ribosome biogenesis in eukaryotes
Ribosome
Protein processing in endoplasmic reticulum
Proteasome
Phagosome
Parkinsons disease
Oxidative phosphorylation
N−Glycan biosynthesis
Lysosome
Influenza A
Huntingtons disease
Herpes simplex infection
Epstein−Barr virus infection
Endocytosis
Antigen processing and presentation
Alzheimers disease
0.3
0.4
0.5
0.6
0.7
0.8
Gene number
30
40
50
60
70
c
Native endothelial cells vs Native fibroblasts
Vibrio cholerae infection
Small cell lung cancer
Ribosome
Protein processing in endoplasmic reticulum
Proteasome
Prostate cancer
Pathways in cancer
Parkinsons disease
Oxidative phosphorylation
NOD−like receptor signaling pathway
Huntingtons disease
GnRH signaling pathway
Glioma
Gap junction
Epstein−Barr virus infection
Epithelial cell signaling in Helicobacter pylori infection
Circadian rhythm
Chronic myeloid leukemia
Chemokine signaling pathway
Alzheimers disease
0.2
0.3
RichFactor
Gene number
10
20
30
40
Qvalue
1.00
0.50
0.75
0.25
0.00

Supplemental Figure 6: Top 20 statistics of KEGG pathway enrichment after RNA-seq assay in Figure 3 by comparing with native fibroblasts

(a) Venn diagrams of mRNA significantly upregulated or downregulated in MEndoT-derived cells and native endothelial cells compared with native fibroblasts. (b-c) Top 20 statistics of KEGG pathway enrichment after RNA-seq assay in Figure 3 by comparing with native fibroblasts, red font represents the pathways that are enriched in both MEndoT-derived cells and native endothelial cells compared with native fibroblasts.

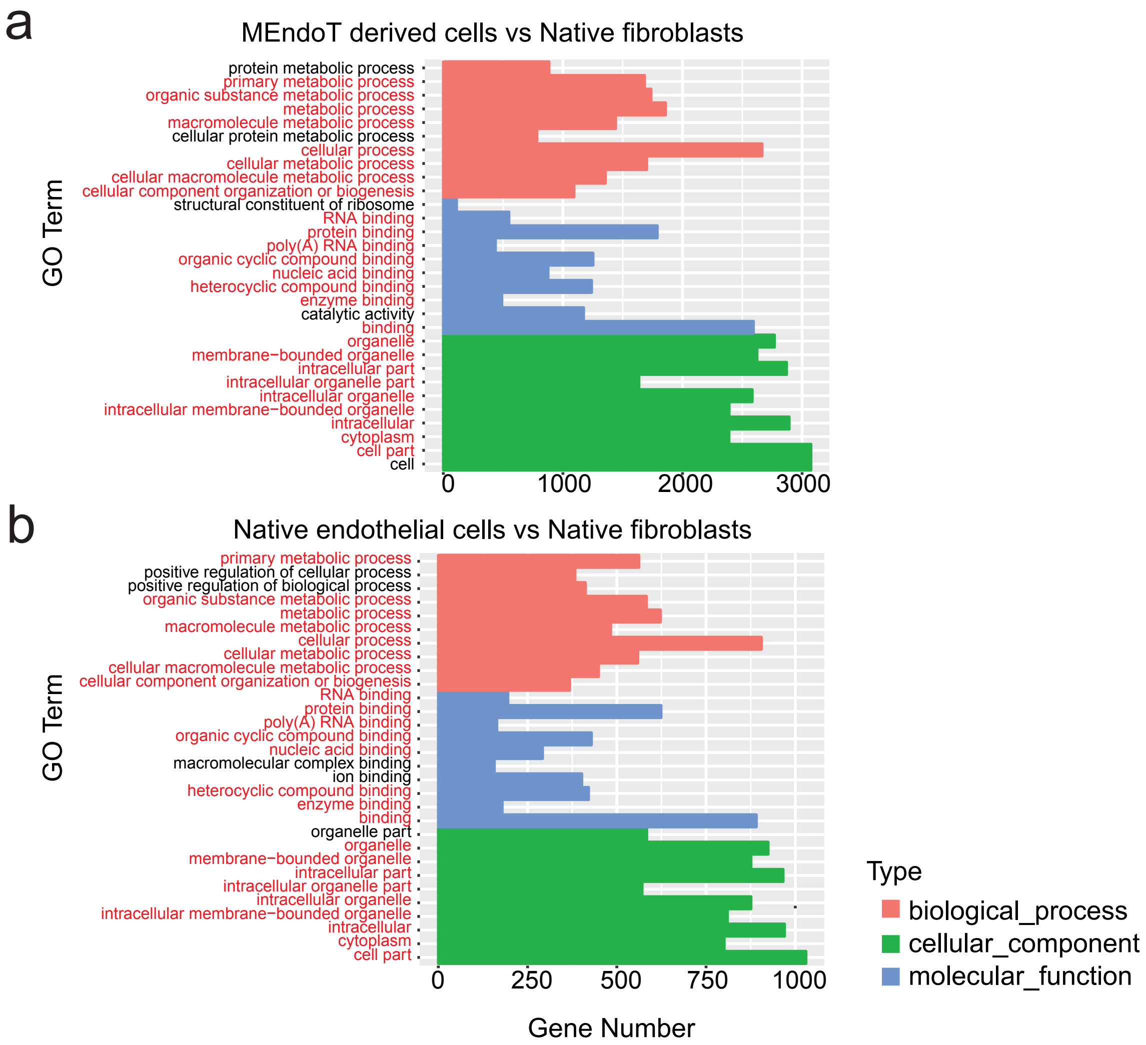

Supplemental Figure 7: Most enriched GO results of three ontologies after RNA-seq assay in Figure 3 by comparing with native fibroblasts

Red font represents the biological processes that were enriched in both MEndoT-derived cells and native endothelial cells on comparing with native fibroblasts.

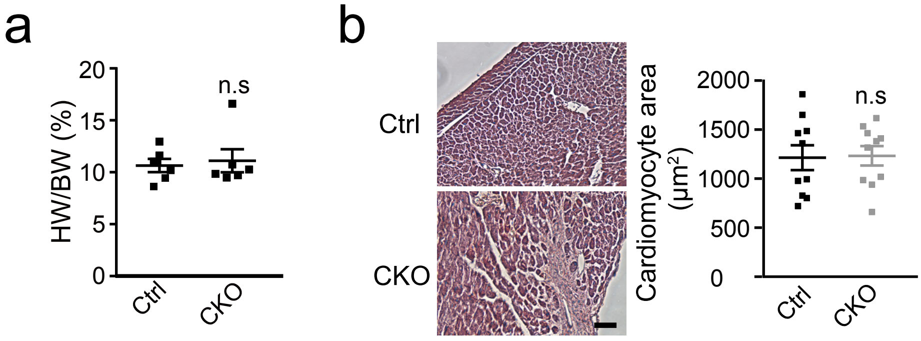

Supplemental Figure 8: Ratio of heart to body weight and cardiomyocyte size in Ctrl and CKO mice 28 days post-TAC/Sham

(a) Ratio of heart to body weight (n=6 animals/group). (b) Quantitation of cardiomyocyte size in H&E stained heart tissue (n=10 animals/group). Graphs show means±S.E.M.; *p<0.05,using ANOVA with post hoc tests. Scale bar: 50 μm.

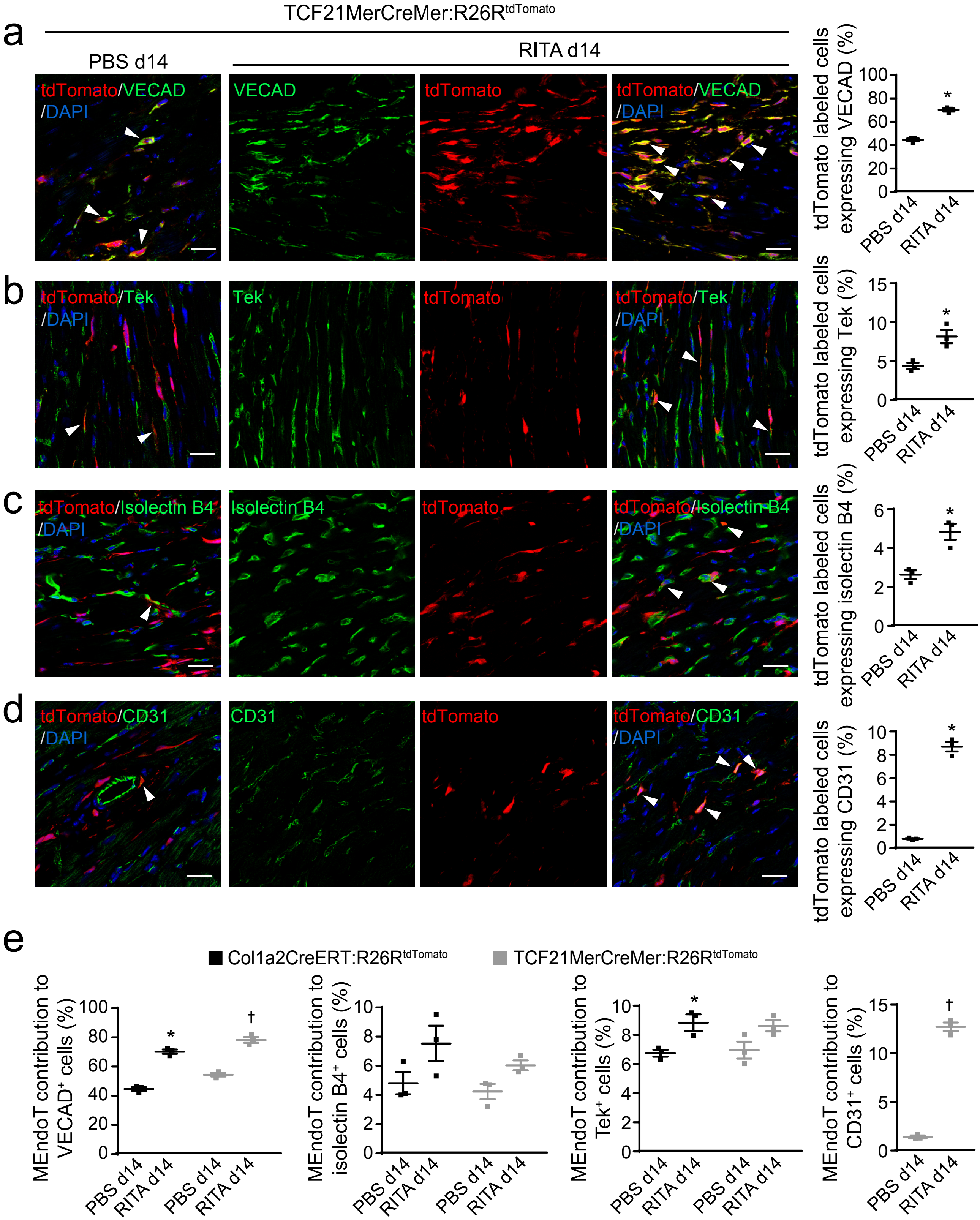
a
TCF21MerCreMer:R26R tdTomato
PBS d14
RITA d14
tdTomato/VECAD/DAPI
VECAD
tdTomato
tdTomato labeled cells expressing VECAD (%)
100
80
60
40
20
0
PBS d14
RITA d14
*
b
tdTomato/Tek/DAPI
Tek
tdTomato labeled cells expressing Tek (%)
15
10
5
0
c
tdTomato/Isolectin B4/DAPI
Isolectin B4
tdTomato labeled cells expressing isolectin B4 (%)
6
4
2
0
d
tdTomato/CD31/DAPI
CD31
tdTomato labeled cells expressing CD31 (%)
10
8
6
4
2
0
e
Col1a2CreERT:R26R tdTomato
TCF21MerCreMer:R26R tdTomato
MEndoT contribution to VECAD⁺ cells (%)
MEndoT contribution to isolectin B4⁺ cells (%)
MEndoT contribution to Tek⁺ cells (%)
MEndoT contribution to CD31⁺ cells (%)
†

Supplemental Figure 9: Immunofluorescence staining for endothelial markers in the heart of TCF21-MerCreMer: R26R^{tdTomato} mice treated with PBS or RITA for 14 days post TAC

(a-d) Immunofluorescence staining images and percentage of labeled cardiac fibroblasts expressing endothelial markers. (e) MEndoT contribution to corresponding endothelial marker positive cells. All graphs show mean±S.E.M. n=3 animals, *$p<0.05$ using an unpaired t-test compared with PBS control. Colocalization of fluorophores is indicated by the arrowhead. Scale bar: 25 μm.

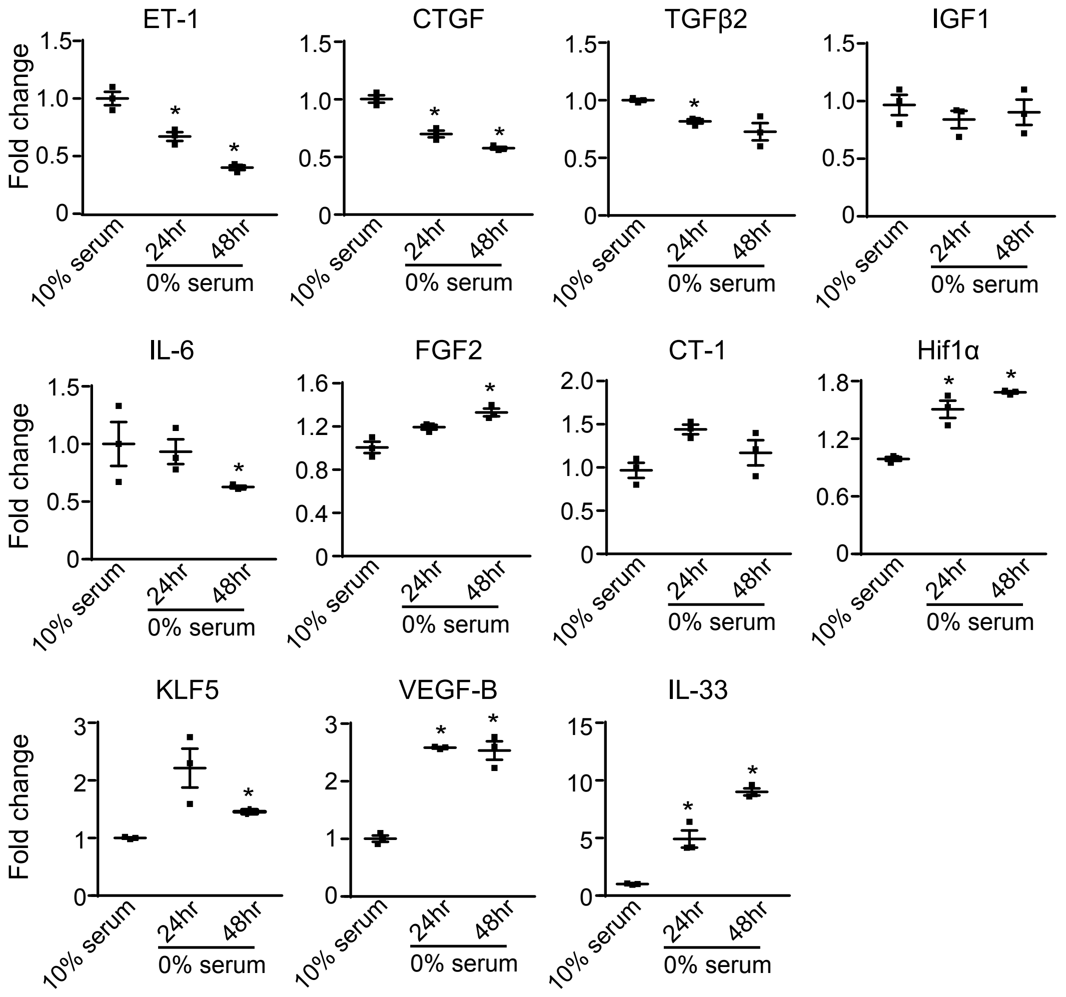

Supplemental Figure 10: qPCR results for Figure 7b

The expression of paracrine factors in fibroblasts undergoing MEndoT under serum starvation as determined by qPCR. These paracrine factors have been demonstrated to be important for cardiac hypertrophy and heart failure. All graphs show mean±S.E.M.n=3 experiment repeat, *p<0.05 using an unpaired t-test compared with fibroblasts in 10% serum.

Supplemental table 1:Gene expression heatmap of 43 gene indicative of fibroblast lineage commitment in Figure 3c and Supplemental Figure 5c:

	MEndoT derived cells VS Native fibroblasts					q_value<0.05
GeneID	GeneName	fold change	up-or-down	p_value	q_value	Description
ENSMUSG00000031740	Mmp2	-6.84982	down	0.00005	0.000300251	matrix metallopeptidase 2 [Source:MGI Symbol;Acc:MGI:97009]
ENSMUSG00000029061	Mmp23	#NAME?	down	0.0001	0.000563679	matrix metallopeptidase 23 [Source:MGI Symbol;Acc:MGI:1347361]
ENSMUSG00000020241	Col6a2	#NAME?	down	0.00005	0.000300251	collagen, type VI, alpha 2 [Source:MGI Symbol;Acc:MGI:88460]
ENSMUSG00000029661	Col1a2	#NAME?	down	0.00005	0.000300251	collagen, type I, alpha 2 [Source:MGI Symbol;Acc:MGI:88468]
ENSMUSG00000057967	Fgf18	#NAME?	down	0.00005	0.000300251	fibroblast growth factor 18 [Source:MGI Symbol;Acc:MGI:1277980]
ENSMUSG00000076431	Sox4	-3.39886	down	0.00005	0.000300251	SRY (sex determining region Y)-box 4 [Source:MGI Symbol;Acc:MGI:98366]
ENSMUSG00000029231	Pdgfra	#NAME?	down	0.00005	0.000300251	platelet derived growth factor receptor, alpha polypeptide [Source:MGI Symbol;Acc:MGI:97530]
ENSMUSG00000031230	Fgf16	#NAME?	down	0.00005	0.000300251	fibroblast growth factor 16 [Source:MGI Symbol;Acc:MGI:1931627]
ENSMUSG00000000567	Sox9	#NAME?	down	0.00005	0.000300251	SRY (sex determining region Y)-box 9 [Source:MGI Symbol;Acc:MGI:98371]
ENSMUSG00000037225	Fgf2	#NAME?	down	0.0001	0.000563679	fibroblast growth factor 2 [Source:MGI Symbol;Acc:MGI:95516]
ENSMUSG00000020170	Frs2	-3.65964	down	0.002	0.00781613	fibroblast growth factor receptor substrate 2 [Source:MGI Symbol;Acc:MGI:1100860]
ENSMUSG00000047632	Fgfbp3	#NAME?	down	0.0001	0.000563679	fibroblast growth factor binding protein 3 [Source:MGI Symbol;Acc:MGI:1919764]
ENSMUSG00000068196	Col8a1	-6.62312	down	0.00005	0.000300251	collagen, type VIII, alpha 1 [Source:MGI Symbol;Acc:MGI:88463]
ENSMUSG00000008090	Fgfrl1	#NAME?	down	0.00005	0.000300251	fibroblast growth factor receptor-like 1 [Source:MGI Symbol;Acc:MGI:2150920]
ENSMUSG00000001506	Col1a1	#NAME?	down	0.00005	0.000300251	collagen, type I, alpha 1 [Source:MGI Symbol;Acc:MGI:88467]
ENSMUSG00000064080	Fbln2	-7.70733	down	0.00005	0.000300251	fibulin 2 [Source:MGI Symbol;Acc:MGI:95488]
ENSMUSG00000059173	Pde1a	#NAME?	down	0.00005	0.000300251	phosphodiesterase 1A, calmodulin-dependent [Source:MGI Symbol;Acc:MGI:1201792]
ENSMUSG00000001119	Col6a1	#NAME?	down	0.00005	0.000300251	collagen, type VI, alpha 1 [Source:MGI Symbol;Acc:MGI:88459]
ENSMUSG00000026674	Ddr2	#NAME?	down	0.00005	0.000300251	discoidin domain receptor family, member 2 [Source:MGI Symbol;Acc:MGI:1345277]
ENSMUSG00000017737	Mmp9	inf	up	0.00005	0.000300251	matrix metallopeptidase 9 [Source:MGI Symbol;Acc:MGI:97011]
ENSMUSG00000049723	Mmp12	inf	up	0.00005	0.000300251	matrix metallopeptidase 12 [Source:MGI Symbol;Acc:MGI:97005]
ENSMUSG00000018593	Sparc	6.0976	up	0.0004	0.00194309	secreted acidic cysteine rich glycoprotein [Source:MGI Symbol;Acc:MGI:98373]
ENSMUSG00000032011	Thy1	9.36331	up	0.00335	0.012205	thymus cell antigen 1, theta [Source:MGI Symbol;Acc:MGI:98747]
ENSMUSG00000026728	Vim	4.91292	up	0.00005	0.000300251	vimentin [Source:MGI Symbol;Acc:MGI:98932]
ENSMUSG00000031375	Bgn	6.79899	up	0.00005	0.000300251	biglycan [Source:MGI Symbol;Acc:MGI:88158]
ENSMUSG00000026193	Fn1	10.0131	up	0.00065	0.00296093	fibronectin 1 [Source:MGI Symbol;Acc:MGI:95566]
	MEndoT derived cells VS Native fibroblasts					q_value>0.05
GeneID	GeneName	fold change	up-or-down	p_value	q_value	Description
ENSMUSG00000016458	Wt1	inf	up	0.0178	0.0502728	Wilms tumor 1 homolog [Source:MGI Symbol;Acc:MGI:98968]
ENSMUSG00000031503	Col4a2	3.17938	up	0.311	0.354585	collagen, type IV, alpha 2 [Source:MGI Symbol;Acc:MGI:88455]
ENSMUSG00000024620	Pdgfrb	7.63417	up	0.05725	0.126823	platelet derived growth factor receptor, beta polypeptide [Source:MGI Symbol;Acc:MGI:97531]
ENSMUSG00000026208	Des	12.6046	up	0.1914	0.275308	desmin [Source:MGI Symbol;Acc:MGI:94885]
ENSMUSG00000026879	Gsn	2.32256	up	0.1432	0.226388	gelsolin [Source:MGI Symbol;Acc:MGI:95851]
ENSMUSG00000040152	Thbs1	0.551917	up	0.6911	0.713319	thrombospondin 1 [Source:MGI Symbol;Acc:MGI:98737]
ENSMUSG00000001020	S100a4	8.27903	up	0.11875	0.201243	S100 calcium binding protein A4 [Source:MGI Symbol;Acc:MGI:1330282]
ENSMUSG00000043613	Mmp3	2.47099	up	0.27055	0.320212	matrix metallopeptidase 3 [Source:MGI Symbol;Acc:MGI:97010]
ENSMUSG00000027750	Postn	6.79878	up	0.25885	0.311576	periostin, osteoblast specific factor [Source:MGI Symbol;Acc:MGI:1926321]
ENSMUSG00000031502	Col4a1	4.61744	up	0.0221	0.0600519	collagen, type IV, alpha 1 [Source:MGI Symbol;Acc:MGI:88454]
ENSMUSG00000021669	Col4a3bp	-2.30927	down	0.014	0.0411956	collagen, type IV, alpha 3 (Goodpasture antigen) binding protein [Source:MGI Symbol;Acc:MGI:1915268]
ENSMUSG00000070643	Sox13	-1.19902	down	0.37065	0.410624	SRY (sex determining region Y)-box 13 [Source:MGI Symbol;Acc:MGI:98361]
ENSMUSG00000032419	Tbx18	-20.8445	down	0.2449	0.307089	T-box18 [Source:MGI Symbol;Acc:MGI:1923615]
ENSMUSG00000045680	Tcf21	-6.95584	down	0.17595	0.260152	transcription factor 21 [Source:MGI Symbol;Acc:MGI:1202715]
ENSMUSG00000026042	Col5a2	-5.54129	down	0.10195	0.184747	collagen, type V, alpha 2 [Source:MGI Symbol;Acc:MGI:88458]
ENSMUSG00000019929	Dcn	-6.74172	down	0.04	0.096544	decorin [Source:MGI Symbol;Acc:MGI:94872]
ENSMUSG00000040249	Lrp1	-4.86259	down	0.22	0.29875	low density lipoprotein receptor-related protein 1 [Source:MGI Symbol;Acc:MGI:96828]

Note: "#NAME?"=-∞, "inf"=+∞.

Supplemental table 2:Gene expression heatmap of 60 gene indicative of endothelial lineage commitment in Figure 3d and Supplemental Figure 5d

		MEndoT derived cells VS Native fibroblasts					Native endothelial cells VS Native fibroblasts				MEndoT derived cells VS Native fibroblasts q_value<0.05 or Native endothelial cells VS Native fibroblasts q_value<0.05
GeneID	GeneName	fold change	up-or-down	p_value	q_value		fold change	up-or-down	p_value	q_value	Description
ENSMUSG00000033191	Tie1	0					inf	up	0.00005	0.00119258	tyrosine kinase with immunoglobulin-like and EGF-like domains 1 [Source:MGI Symbol;Acc:MGI:99906]
ENSMUSG00000041378	Cldn5	0					inf	up	0.00005	0.00119258	claudin 5 [Source:MGI Symbol;Acc:MGI:1276112]
ENSMUSG00000027962	Vcam1	-2.25985	down	0.09215	0.174731		-1.451880058	down	0.00075	0.0110068	vascular cell adhesion molecule 1 [Source:MGI Symbol;Acc:MGI:98926]
ENSMUSG00000031520	Vegfc	#NAME?	down	0.00005	0.000300251		-2.449787072	down	0.00005	0.00119258	vascular endothelial growth factor C [Source:MGI Symbol;Acc:MGI:109124]
ENSMUSG00000033420	Antxr1	#NAME?	down	0.00005	0.000300251		0.504812261	up	0.54455	0.725422	anthrax toxin receptor 1 [Source:MGI Symbol;Acc:MGI:1916788]
ENSMUSG00000024140	Epas1	#NAME?	down	0.00005	0.000300251		0.448125531	up	0.3649	0.586908	endothelial PAS domain protein 1 [Source:MGI Symbol;Acc:MGI:109169]
ENSMUSG00000028073	Pear1	0.168051	up	0.89715	0.90506		-2.187816502	down	0.0034	0.0340424	platelet endothelial aggregation receptor 1 [Source:MGI Symbol;Acc:MGI:1920432]
ENSMUSG00000074743	Thbd	0					-0.461744649	down	0.058	0.221655	thrombomodulin [Source:MGI Symbol;Acc:MGI:98736]
ENSMUSG00000037405	Icam1	0					-0.782120057	down	0.00275	0.0296594	intercellular adhesion molecule 1 [Source:MGI Symbol;Acc:MGI:96392]
ENSMUSG00000062515	Fabp4	12.7582	up	0.00005	0.000300251		7.491103543	up	0.00005	0.00119258	fatty acid binding protein 4, adipocyte [Source:MGI Symbol;Acc:MGI:88038]
ENSMUSG00000054690	Emcn	inf	up	0.00005	0.000300251		inf	up	0.00005	0.00119258	endomucin [Source:MGI Symbol;Acc:MGI:1891716]
ENSMUSG00000045382	Cxcr4	inf	up	0.003	0.011061		inf	up	0.00005	0.00119258	chemokine (C-X-C motif) receptor 4 [Source:MGI Symbol;Acc:MGI:109563]
ENSMUSG00000020717	Pecam1(CD31)	inf	up	0.00115	0.0048541		inf	up	0.00005	0.00119258	platelet/endothelial cell adhesion molecule 1 [Source:MGI Symbol;Acc:MGI:97537]
ENSMUSG00000022122	Ednrb	inf	up	0.00155	0.00628829		inf	up	0.00005	0.00119258	endothelin receptor type B [Source:MGI Symbol;Acc:MGI:102720]
ENSMUSG00000002944	Cd36	inf	up	0.00005	0.000300251		inf	up	0.00005	0.00119258	CD36 antigen [Source:MGI Symbol;Acc:MGI:107899]
ENSMUSG00000032135	Mcam	inf	up	0.00425	0.0149619		inf	up	0.04005	0.177683	melanoma cell adhesion molecule [Source:MGI Symbol;Acc:MGI:1933966]
ENSMUSG00000026921	Egfl7	inf	up	0.00005	0.000300251		inf	up	0.00005	0.00119258	EGF-like domain 7 [Source:MGI Symbol;Acc:MGI:2449923]
ENSMUSG00000026814	Eng	4.51332	up	0.0651	0.138891		-1.450430853	down	0.00005	0.00119258	endoglin [Source:MGI Symbol;Acc:MGI:95392]
ENSMUSG00000030341	Tnfrsf1a	4.99024	up	0.0089	0.0281115		-0.670126173	down	0.0229	0.127117	tumor necrosis factor receptor superfamily, member 1a [Source:MGI Symbol;Acc:MGI:1314884]
ENSMUSG00000048163	Selplg	inf	up	0.00005	0.000300251		0				selectin, platelet (p-selectin) ligand [Source:MGI Symbol;Acc:MGI:106689]
ENSMUSG00000017309	Cd300lg	inf	up	0.006	0.0202415		0				CD300 molecule like family member G [Source:MGI Symbol;Acc:MGI:1289168]
ENSMUSG00000009687	Fxyd5	7.11533	up	0.00005	0.000300251		0.393864575	up	0.08145	0.271134	FXYD domain-containing ion transport regulator 5 [Source:MGI Symbol;Acc:MGI:1201785]
ENSMUSG00000028583	Pdpn	3.65994	up	0.0026	0.00979783		0.222619691	up	0.26915	0.499739	podoplanin [Source:MGI Symbol;Acc:MGI:103098]
ENSMUSG00000025510	Cd151	8.35824	up	0.00005	0.000300251		1.872853547	up	0.00005	0.00119258	CD151 antigen [Source:MGI Symbol;Acc:MGI:1096360]
ENSMUSG00000007655	Cav1	10.3306	up	0.00005	0.000300251		2.565332528	up	0.00005	0.00119258	caveolin 1, caveolae protein [Source:MGI Symbol;Acc:MGI:102709]
ENSMUSG00000027533	Fabp5	6.63764	up	0.1848	0.269349		1.289178708	up	0.00225	0.0259426	fatty acid binding protein 5, epidermal [Source:MGI Symbol;Acc:MGI:101790]
ENSMUSG00000015092	Edf1	6.80655	up	0.00005	0.000300251		0.939868774	up	0.00065	0.0099481	endothelial differentiation-related factor 1 [Source:MGI Symbol;Acc:MGI:1891227]

		MEndoT derived cells VS Native fibroblasts					Native endothelial cells VS Native fibroblasts				MEndoT derived cells VS Native fibroblasts q_value>0.05 and Native endothelial cells VS Native fibroblasts q_value>0.05
GeneID	GeneName	fold change	up-or-down	p_value	q_value		fold change	up-or-down	p_value	q_value	Description
ENSMUSG00000003032	Klf4	-1.96011	down	0.2998	0.344482		1.54640789	up	0.09575	0.298777	Kruppel-like factor 4 (gut) [Source:MGI Symbol;Acc:MGI:1342287]
ENSMUSG00000026034	Clk1	-0.566601	down	0.4343	0.47024		1.654368066	up	0.00015	0.030707	CDC-like kinase 1 [Source:MGI Symbol;Acc:MGI:107403]
ENSMUSG00000042286	Stab1	0					3.497865134	up	0.0957	0.298694	stabilin 1 [Source:MGI Symbol;Acc:MGI:2178742]
ENSMUSG00000031380	Vegfd	0					1.798585773	up	0.5835	0.751576	vascular endothelial growth factor D [Source:MGI Symbol;Acc:MGI:108037]
ENSMUSG00000006386	Tek	0					1.313739117	up	0.32205	0.549545	endothelial-specific receptor tyrosine kinase [Source:MGI Symbol;Acc:MGI:98664]
ENSMUSG00000026580	Selp	0					inf	up	0.0603	0.226937	selectin, platelet [Source:MGI Symbol;Acc:MGI:98280]
ENSMUSG00000071042	Rasgrp3	0					4.681335726	up	0.2341	0.472787	RAS, guanyl releasing protein 3 [Source:MGI Symbol;Acc:MGI:3028579]
ENSMUSG00000028978	Nos3	0					5.106105781	up	0.2746	0.504611	nitric oxide synthase 3, endothelial cell [Source:MGI Symbol;Acc:MGI:97362]
ENSMUSG00000027079	Clp1	-1.28665	down	0.10435	0.187235		-0.861308523	down	0.2125	0.453729	CLP1, cleavage and polyadenylation factor I subunit [Source:MGI Symbol;Acc:MGI:2138968]
ENSMUSG00000027611	Procr	-8.31618	down	0.2404	0.306267		0.351479793	up	0.1616	0.39022	protein C receptor, endothelial [Source:MGI Symbol;Acc:MGI:104596]
ENSMUSG00000023951	Vegfa	-8.59065	down	0.2086	0.291249		-0.999842741	down	0.03425	0.161289	vascular endothelial growth factor A [Source:MGI Symbol;Acc:MGI:103178]
ENSMUSG00000028599	Tnfrsf1b	0.163003	up	0.86735	0.877054		-2.801524126	down	0.01165	0.0817089	tumor necrosis factor receptor superfamily, member 1b [Source:MGI Symbol;Acc:MGI:1314883]
ENSMUSG00000022615	Tymp	0					-0.485813653	down	0.81325	0.891089	thymidine phosphorylase [Source:MGI Symbol;Acc:MGI:1920212]
ENSMUSG00000030123	Plxnd1	0					-0.265165966	down	0.74115	0.849369	plexin D1 [Source:MGI Symbol;Acc:MGI:2154244]
ENSMUSG00000062300	Nectin2	7.403	up	0.07995	0.159677		-8.857156766	down	0.14165	0.361822	nectin cell adhesion molecule 2 [Source:MGI Symbol;Acc:MGI:97822]
ENSMUSG00000026582	Sele	2.68715	up	0.2099	0.292072		-2.681202128	down	0.2042	0.444647	selectin, endothelial cell [Source:MGI Symbol;Acc:MGI:98278]
ENSMUSG00000056481	Cd248	7.30869	up	0.0657	0.139811		-6.135052176	down	0.1928	0.431518	CD248 antigen, endosialin [Source:MGI Symbol;Acc:MGI:1917695]
ENSMUSG00000031871	Cdh5(VECAD)	12.9284	up	0.449	0.483964		10.02292438	up	0.25165	0.483012	cadherin 5 [Source:MGI Symbol;Acc:MGI:105057]
ENSMUSG00000029648	Flt1	15.0319	up	0.3203	0.362728		11.84018753	up	0.24055	0.475844	FMS-like tyrosine kinase 1 [Source:MGI Symbol;Acc:MGI:95558]
ENSMUSG00000062960	Kdr	15.012	up	0.2529	0.308768		7.212827406	up	0.2374	0.474089	kinase insert domain protein receptor [Source:MGI Symbol;Acc:MGI:96683]
ENSMUSG00000001946	Esam	11.6957	up	0.02835	0.073595		6.763461647	up	0.01495	0.0977108	endothelial cell-specific adhesion molecule [Source:MGI Symbol;Acc:MGI:1916774]
ENSMUSG00000073599	Ecscr	10.1084	up	0.28105	0.328589		6.014833842	up	0.2131	0.454167	endothelial cell surface expressed chemotaxis and apoptosis regulator [Source:MGI Symbol;Acc:MGI:1915795]
ENSMUSG00000001029	Icam2	11.8618	up	0.08675	0.168138		6.182975452	up	0.0493	0.200506	intercellular adhesion molecule 2 [Source:MGI Symbol;Acc:MGI:96394]
ENSMUSG00000074874	Ctla2b	13.3639	up	0.2882	0.334838		7.491912726	up	0.2465	0.479281	cytotoxic T lymphocyte-associated protein 2 beta [Source:MGI Symbol;Acc:MGI:88555]
ENSMUSG00000027435	Cd93	11.0135	up	0.3747	0.414343		6.009318964	up	0.1032	0.310829	CD93 antigen [Source:MGI Symbol;Acc:MGI:106664]
ENSMUSG00000022074	Tnfrsf10b	2.22486	up	0.1749	0.259491		-0.215363839	down	0.55715	0.73396	tumor necrosis factor receptor superfamily, member 10b [Source:MGI Symbol;Acc:MGI:1341090]
ENSMUSG00000016494	Cd34	0.557327	up	0.6831	0.705468		-0.126873978	down	0.63225	0.782612	CD34 antigen [Source:MGI Symbol;Acc:MGI:88329]
ENSMUSG00000002396	Ocel1	8.76826	up	0.09135	0.173735		-1.545049279	down	0.3718	0.593477	occludin/ELL domain containing 1 [Source:MGI Symbol;Acc:MGI:1924340]
ENSMUSG00000025608	Podxl	3.97962	up	0.242	0.306511		1.063008545	up	0.06805	0.245099	podocalyxin-like [Source:MGI Symbol;Acc:MGI:1351317]
ENSMUSG00000000058	Cav2	7.14441	up	0.1708	0.256057		1.387668778	up	0.0761	0.260544	caveolin 2 [Source:MGI Symbol;Acc:MGI:107571]
ENSMUSG00000042379	Esm1	3.36418	up	0.14595	0.22932		0.329834748	up	0.3606	0.583275	endothelial cell-specific molecule 1 [Source:MGI Symbol;Acc:MGI:1918940]
ENSMUSG00000035107	Dcbld2	5.07638	up	0.07045	0.146413		0.078867259	up	0.88705	0.933982	discoidin, CUB and LCCL domain containing 2 [Source:MGI Symbol;Acc:MGI:1920629]
ENSMUSG00000038304	Cd160	8.67832	up	0.0412	0.0986873		0				CD160 antigen [Source:MGI Symbol;Acc:MGI:1860383]

Note: "#NAME?"=-∞, "inf"=+∞.

Supplemental table 3: Gene expression heatmap of 9 gene of paracrine factor in Figure 7a

	**MEndoT derived cells VS Native fibroblasts**					
GeneID	GeneName	fold change	up-or-down	p_value	q_value	Description
ENSMUSG00000024810	IL-33	7.79222	up	0.0992	0.182033	interleukin 33 [Source:MGI Symbol;Acc:MGI:1924375]
ENSMUSG00000021109	Hif1a	1.69921	up	0.10885	0.191507	hypoxia inducible factor 1, alpha subunit [Source:MGI Symbol;Acc:MGI:106918]
ENSMUSG00000005148	KLF5	#NAME?	down	5.00E-05	0.000300251	Kruppel-like factor 5 [Source:MGI Symbol;Acc:MGI:1338056]
ENSMUSG00000039239	TGFβ2	-0.330866	down	0.7731	0.789769	transforming growth factor, beta 2 [Source:MGI Symbol;Acc:MGI:98726]
ENSMUSG00000040663	Clcf1	-3.30881	down	0.1996	0.283168	cardiotrophin-like cytokine factor 1 [Source:MGI Symbol;Acc:MGI:1930088]
ENSMUSG00000020053	IGF1	#NAME?	down	5.00E-05	0.000300251	insulin-like growth factor 1 [Source:MGI Symbol;Acc:MGI:96432]
ENSMUSG00000025746	IL-6	#NAME?	down	5.00E-05	0.000300251	interleukin 6 [Source:MGI Symbol;Acc:MGI:96559]
ENSMUSG00000037225	FGF2	#NAME?	down	0.0001	0.000563679	fibroblast growth factor 2 [Source:MGI Symbol;Acc:MGI:95516]
ENSMUSG00000034394	LIF	-3.43397	down	0.00025	0.00127489	leukemia inhibitory factor [Source:MGI Symbol;Acc:MGI:96787]

Note: "#NAME?"=-∞, "inf"=+∞.
